# Three-Dimensional Portable Document Format (3D PDF) in Clinical Communication and Biomedical Sciences: Systematic Review of Applications, Tools, and Protocols

**DOI:** 10.2196/10295

**Published:** 2018-08-07

**Authors:** Axel Newe, Linda Becker

**Affiliations:** ^1^ Chair of Medical Informatics Friedrich-Alexander University Erlangen-Nürnberg Erlangen Germany; ^2^ NewTec GmbH Pfaffenhofen an der Roth Germany; ^3^ Chair of Health Psychology Friedrich-Alexander University Erlangen-Nürnberg Erlangen Germany

**Keywords:** 3D PDF, 3D visualization, interactive, clinical communication, biomedical science, tools, protocols, apps, online data sharing, scholarly publishing, electronic publishing

## Abstract

**Background:**

The Portable Document Format (PDF) is the standard file format for the communication of biomedical information via the internet and for electronic scholarly publishing. Although PDF allows for the embedding of three-dimensional (3D) objects and although this technology has great potential for the communication of such data, it is not broadly used by the scientific community or by clinicians.

**Objective:**

The objective of this review was to provide an overview of existing publications that apply 3D PDF technology and the protocols and tools for the creation of model files and 3D PDFs for scholarly purposes to demonstrate the possibilities and the ways to use this technology.

**Methods:**

A systematic literature review was performed using PubMed and Google Scholar. Articles searched for were in English, peer-reviewed with biomedical reference, published since 2005 in a journal or presented at a conference or scientific meeting. Ineligible articles were removed after screening. The found literature was categorized into articles that (1) applied 3D PDF for visualization, (2) showed ways to use 3D PDF, and (3) provided tools or protocols for the creation of 3D PDFs or necessary models. Finally, the latter category was analyzed in detail to provide an overview of the state of the art.

**Results:**

The search retrieved a total of 902 items. Screening identified 200 in-scope publications, 13 covering the use of 3D PDF for medical purposes. Only one article described a clinical routine use case; all others were pure research articles. The disciplines that were covered beside medicine were many. In most cases, either animal or human anatomies were visualized. A method, protocol, software, library, or other tool for the creation of 3D PDFs or model files was described in 19 articles. Most of these tools required advanced programming skills and/or the installation of further software packages. Only one software application presented an all-in-one solution with a graphical user interface.

**Conclusions:**

The use of 3D PDF for visualization purposes in clinical communication and in biomedical publications is still not in common use, although both the necessary technique and suitable tools are available, and there are many arguments in favor of this technique. The potential of 3D PDF usage should be disseminated in the clinical and biomedical community. Furthermore, easy-to-use, standalone, and free-of-charge software tools for the creation of 3D PDFs should be developed.

## Introduction

### Background

**Figure 1:**
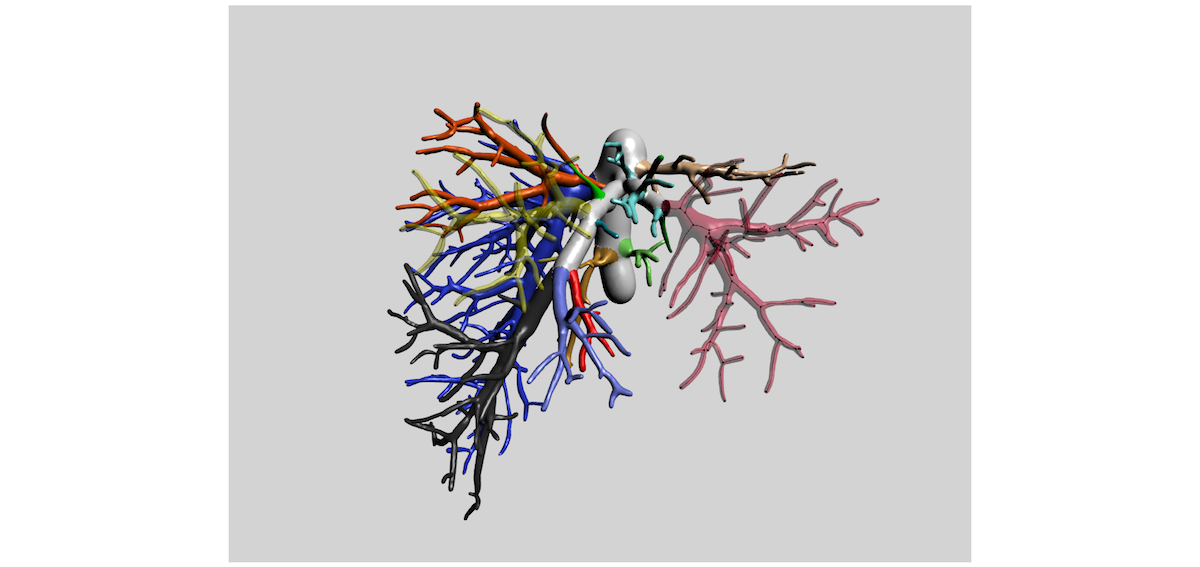
Vessel tree of a liver. See Multimedia Appendix 1 for 3D PDF version of this figure and Multimedia Appendix 2 for a 3D PDF version of this entire article.

The best-known and most widely used data format for the exchange of electronic documents is probably the Portable Document Format (PDF), which was standardized by the International Organization for Standardization (ISO) as ISO 32000-2:2017 [1]. Software that can read PDFs is installed on nearly every computer, and most internet browsers and email clients have a built-in PDF renderer. This, in many cases, makes this format the best means for the exchange of electronic documents. However, the PDF offers more features than many people are aware of. Although technically available since 2005, a still lesser-known standard feature of the PDF is the possibility to embed three-dimensional (3D) models, which enables the interactive visualization (eg, zooming, panning, rotating, and selection of components) of such objects with qualified reader software (Figure 1; for an interactive version of this figure, see Multimedia Appendix 1).

A PDF document with embedded 3D objects (3D PDF) has high potential in almost every scenario in which 3D objects should be visualized and exchanged between different platforms (eg, computers with different operating systems). A highly relevant use case is the exchange of medical and biomedical data (eg, the visualization of human anatomy for medical students [2] or clinical data such as the results of surgery planning [3,4]). Therefore, this feature is also considered in the standard AIIM/ASTM BP-01-2008 “Portable Document Format-Healthcare (PDF) A Best Practices Guide” (also known as PDF Healthcare or PDF/H) [5,6]. This standard describes how to use the PDF to exchange and preserve digital health care information in a safe and secure way.

In addition to the exchange between a few persons (eg, a doctor and a patient), 3D PDF offers a very efficient and convenient way for distributing 3D structures through the scientific community by embedding 3D objects into scientific publications.

The primary aim of this review was to investigate the significance of 3D PDF in clinical communication and in scholarly publications in the biomedical sciences. The secondary aim of this review was to provide an overview of the technical possibilities and to present all currently available solutions for creating 3D PDFs and related model files.

### The Portable Document Format

The PDF is a computer file format for the platform-independent definition of electronic documents [1]. It allows for describing electronic documents with preserved fidelity, independently of the software, device, and operating system used to create, display, or print them. Furthermore, PDF is capable of encapsulating all necessary resources (eg, texts, images, or multimedia elements). Along with the open accessibility of the specification, this has led to PDF being the most commonly used file format for the exchange of electronic documents. For example, all scholarly journals allow for an electronic publication of their articles in PDF today.

The PDF was originally developed by Adobe Systems and evolved in the early 1990s from the PostScript page description language for printers. The first specification of PDF was published in 1993; since then, it has been developed further by Adobe until version 1.7 [7], which was released in 2006. This version was given to the ISO, which re-released this specification as ISO standard 32000-1:2008 in July 2008 [8]. The latest version (PDF 2.0) was published in July 2017 [1,9].

The PDF is now the de facto standard for the exchange of electronic documents. The original Adobe Reader software alone has been distributed more than 500 million times around the world [7], and countless other apps for displaying PDF documents are available. For example, Quartz 2D, the native graphics rendering interface for two-dimensional (2D) graphics of the macOS X and iOS operating systems, is based on the PDF specification, (ie, it is an integral part of these operating systems) [10].

### The Development of the Three-Dimensional Portable Document Format

Version 1.6 of the PDF specification, published in 2004 [11], introduced the capability to embed 3D objects, such as those used by software for computer-aided design, into PDF files. At the time of the introduction of this new feature, the format for the data of 3D objects needed to be Universal 3D (U3D), a file format developed by the 3D Industry Forum (which Adobe was part of) as “a single, common, and open 3D standard” and “JPEG for 3D” [12]. U3D is standardized by the European Computer Manufacturers Association (ECMA) as ECMA-363 [13]. The first software to support 3D Artwork (the PDF term for 3D objects in PDF files) was the Adobe Reader 7 (and Adobe Acrobat 7, respectively), published in January 2005. However, the first software that really allowed for an efficient and convenient working with 3D models in PDF was Acrobat 3D, released in early 2006. This version provided tools for the import and conversion of many 3D formats and a 3D editor [14].

In late 2006, Adobe added the ability to embed 3D models in Product Representation Compact (PRC) format into PDF (PDF BaseVersion 1.7, ExtensionLevel 1 [15]), but this feature has not been integrated into the ISO standard 32000-1 from 2008. It took 11 years until the release of PDF 2.0 (32000-2:2017) in July 2017 to formally integrate PRC as 3D format into the PDF standard. However, the support of editing features for 3D was discontinued in later versions of Adobe Acrobat. Acrobat 9 Pro Extended was the last version to include tools for conversion and editing of 3D models. Instead, this functionality has been outsourced to third-party software and has been sold separately since 2010 [16].

Another feature that is notable in this context is the support of JavaScript. This feature was introduced with PDF 1.3 in July 2000 [17] and allows for executing JavaScript commands within a PDF document (eg, after clicking an embedded button or after activating a 3D model).

### Relevance of Three-Dimensional Portable Document Format

Especially in the biomedical domain, the importance of 3D data has grown in recent years (eg, visualization of chemical molecules, anatomy, or vessel systems). With the availability of the necessary technology for the visualization of such 3D data, this technology should consequently be used to avoid a loss of information [18]. This is of particular relevance in scholarly publishing [19]. Furthermore, 3D PDF has a very high potential for the exchange of medical data in clinical communication [3] because it allows for in situ publishing of 3D figures [20].

### Objectives

It has tremendously high potential for clinical communication, many scholarly articles would benefit from interactive visualization of 3D data [19], publishers encourage their authors to use 3D PDF technology [21,22], and some journals provide the necessary tools [23], yet it still does not seem to be common use. Thus, the initial motivation for writing this review paper was to test this hypothesis (ie, that using 3D PDF technology is not common in clinical communication or in biomedical publications). Therefore, as the primary goal, an overview was to be created of the dissemination of 3D PDFs in biomedical publications. The scope was not limited to purely clinical use cases to cover as wide a range of possible apps in all fields of medically relevant research. Furthermore, we will investigate in which research areas and use cases 3D PDF has been applied since the advent of this technology.

After the initial hypothesis was confirmed (200 articles were found over a period of 10 years; see Results), we hypothesized three possible reasons:

1. The existence and/or possibilities of 3D PDF might not be well known among the scientific and clinical community.
2. The necessary knowledge that would enable authors and possible users to actually make use of 3D PDFs might be hard to acquire.
3. The creation of appropriate model files and of final PDF documents might be an overly cumbersome process.

Therefore, the secondary goal of this review was to tackle the previous three issues by providing an overview of (1) existing publications that apply 3D PDF technology to demonstrate its possibilities, (2) available protocols for the creation of 3D PDFs to provide the basic knowledge in compressed form, and (3) existing software tools for the creation of model files and 3D PDFs to show ways to simplify the process.

Although the overview of the existing publications that apply 3D PDF technology in this field may elaborate new ideas of how this form of visualization could augment scholarly communication in biomedicine, the presented protocols and tools can probably be applied in other fields as well.

## Methods

### General Procedure

A systematic literature search was performed in the scientific databases PubMed [24] and Google Scholar [25] to find articles that either applied 3D PDF technology for visualization purposes or articles that generally dealt with the subject of 3D PDF in the context of biomedical sciences.

Because PDF is a vehicle for presenting data rather than an actual research topic, it was likely that a simple database search for this term would not reveal comprehensive coverage of the literature, especially for articles that simply used 3D PDF as a means for visualization. Therefore, a search strategy with two iterations was conceived (see detailed description subsequently). Agreement about this strategy, the inclusion criteria, and the review protocol (see respective sections subsequently) was reached through discussion between all authors.

The first search was performed by AN on March 6, 2017. Three update searches were performed by AN on September 30, 2017, January 1, 2018, and April 30, 2018. The search results were screened and checked for eligibility independently by AN and LB on the basis of predefined inclusion criteria. The remaining articles that were entered into the further analysis were reviewed by means of a predefined review protocol by AN and LB independently.

### Search Strategy

**Figure 2:**
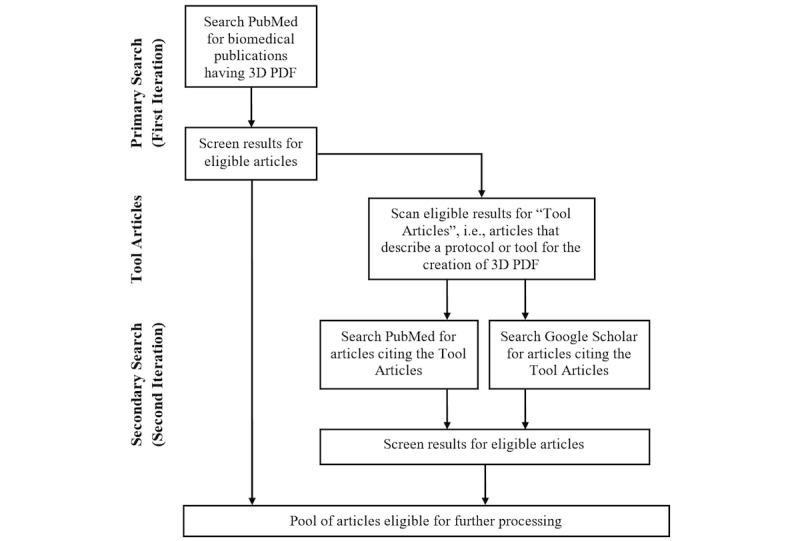
Search strategy.

As mentioned previously, we conceived a two-tiered search strategy (Figure 2). The first iteration was a PubMed search with the following search term: “(((3d) OR (3-d) OR (three dimensional) OR (interactive) OR (surface model)) AND ((pdf) OR (portable document format))) AND (“2005”[Date-Publication]: “3000”[Date-Publication])”.

The goal of this initial search was to reveal all biomedical publications that mentioned 3D PDFs without the most common unwanted meanings for “PDF.” It was limited to articles published after 2004 because 3D PDF technology was not available before 2005.

The results of this first iteration were then screened to exclude articles that did not fulfill the inclusion criteria. The remainders, which will subsequently be referred to as “primary results,” were then analyzed to identify articles that were cited as a source of 3D PDF creation (referred to as “tool articles;” see a more detailed explanation subsequently). Next, both PubMed and Google Scholar were searched for articles that cited these tool articles to identify more articles that applied or mentioned 3D PDF and that could not be found via the initial PubMed search. The results of this second iteration were then also screened for the inclusion criteria and the remaining articles (referred to as “secondary results”) went into the review process along with the primary results and the tool articles.

Finally, a small number of additional articles that were known to us to be eligible, but which were not discovered by the systematic search, were included as well. Further details about the search strategy, the search results, and the screening results are available in Multimedia Appendix 3.

### Inclusion Criteria

The general inclusion criteria for this review were defined as follows: (1) articles from the biomedical domain that applied 3D PDF for visualization or articles that presented protocols or tools for the creation of 3D PDFs or for the creation of 3D PDF-specific, intermediate file formats (U3D or PRC); (2) articles in English language only; and (3) peer-reviewed articles only.

### Review Protocol

To fulfill all these objectives, all eligible articles were first classified into article types using four categories:

1. Application: articles that applied 3D PDF technology for visualization.
2. Descriptive: articles that described a way, method, or idea how to use or to utilize 3D PDF technology.
3. Tool: articles that described a method, protocol, software, library, or other means for the creation of 3D PDFs or model files.
4. Mentioning only: articles that only mentioned the possibility of using 3D PDF (without further details).

The assignment to more than one category (eg, descriptive and application) was allowed.

All articles of the type “application” were then further analyzed for the 3D features used, for the availability of the 3D PDF documents, and for the file size. The following 3D PDF features were considered:

1. Geometry only: articles that only presented the geometry of the 3D model without further features.
2. Multiple colors and transparency: articles that had transparent/colored models.
3. Product Manufacturing Information (PMI)/markup: articles that used PMI features or markup for labeling objects.
4. Scripting: articles that used JavaScript for advanced interactive features.
5. Texture: articles that used textured models.
6. Animation: articles that use animations.

The availability of the 3D PDFs was classified into:

1. Embedded: articles that directly embedded 3D models into the PDF version.
2. Supplement: articles that provided supplemental information files with 3D PDFs.
3. Link to external resources: articles that provided the URL or mail address for accessing the referred 3D PDFs.

Finally, all articles were classified into a scientific discipline (eg, “medicine,” “embryology,” or “human anatomy”; for a full list see Multimedia Appendix 4) and the clinically relevant articles were analyzed further.

## Results

### Search Results

The initial PubMed search retrieved 123 publications; after the third update search, a total of 237 publications were found. After screening these PubMed results, 109 articles were excluded because “PDF” was not used as an acronym for “Portable Document Format,” one was excluded because it was not in English language, one was excluded because it did not cover a biomedical topic, and eight were excluded because they were an erratum only (ie, not a peer-reviewed article). During the full-text eligibility assessment, 65 more articles were excluded because they had no content relevant for 3D PDF. The initial analysis of the remaining 53 primary results revealed 19 tool articles, with 15 of them being duplicates (ie, four new tool articles were identified).

**Figure 3:**
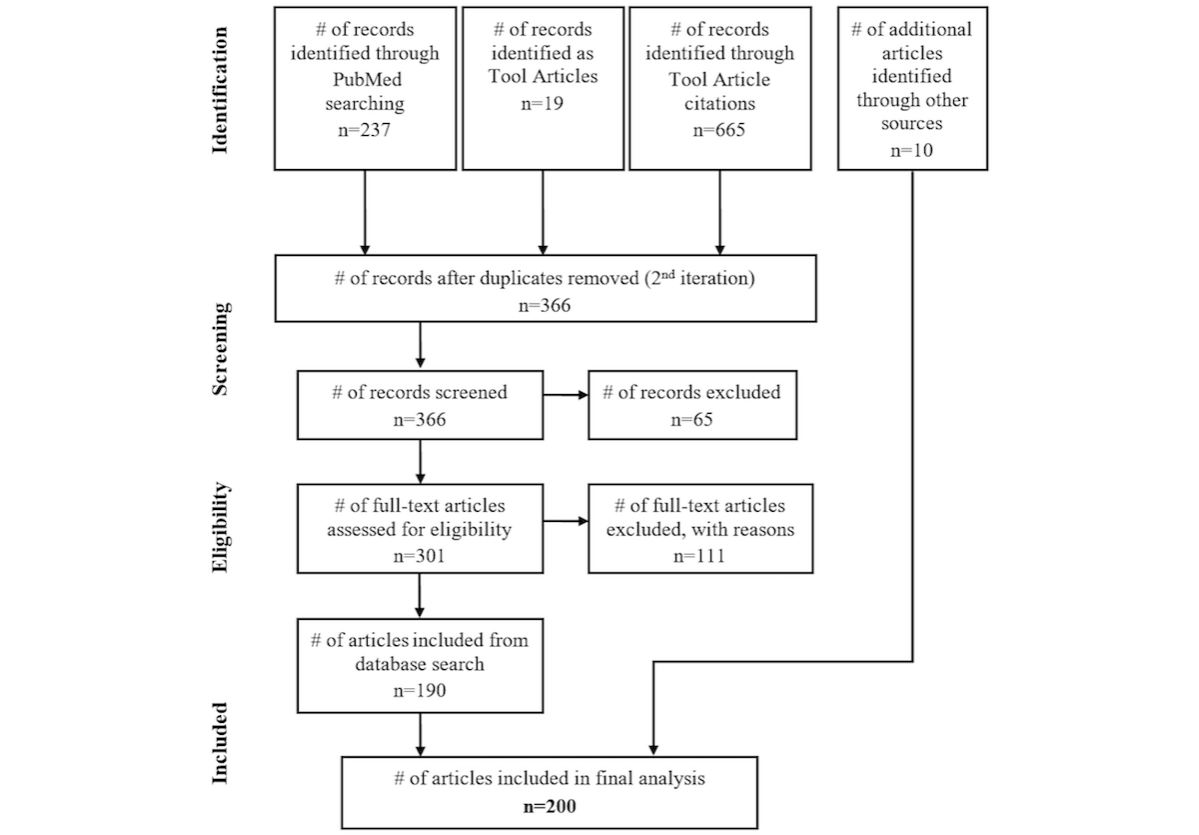
Modified PRISMA flowchart.

The second iteration (ie, the search for articles citing the tool articles) retrieved a total of 128 articles from PubMed and 537 articles from Google Scholar after the third update. After removing 356 duplicates, 366 articles were screened. Of those, 22 were excluded because they were not in the English language and 43 were excluded because they were not peer reviewed. During the full-text eligibility assessment, 48 articles were excluded because they did not fall into the biomedical domain and 63 more articles were excluded because they had no content relevant for 3D PDF. That resulted in 133 articles from the second iteration. Together with the 53 articles of the first iteration, the four additional tool articles, and 10 articles that were included due to our knowledge, 200 articles were included in the final analysis, which is shown in a modified Preferred Reporting Items for Systematic Reviews and Meta-Analyses (PRISMA) flow diagram [26] in Figure 3.

### Overall Analysis Results

Of the total 200 articles, the simple possibility of using 3D PDF (without providing further details) was mentioned in 13 articles (6.5%) [27-39]. Another 18 (9.0%) [2,3,23,40-54] articles described a way, method, or idea how to use or to utilize 3D PDF technology and 19 articles (9.5%) [20,40,55-71] described a method, protocol, software, library, or other means for the creation of 3D PDFs or model files.

In 156 (78.0%) articles, 3D PDF technology was actually applied for the visualization of research results. In 11 (7.1%) more articles, it was claimed that 3D PDF should be available, but no 3D model could be found [58,72-81]. Of the 156 articles with applied 3D PDF technology, 34 (21.8%) [40,55-57,61,64,68,82-108] had the 3D content directly embedded into the PDF of the article, 94 (60.3%) [20,48,59,60,63,66,67,109-195] articles provided the 3D content as supplementary material, and 28 (17.9%) [4,41,62,196-220] referred to an external resource. The 34 articles in which the 3D objects were embedded directly into PDF of the main article were published in 25 different journals.

Different features of 3D PDF were used. In seven articles (4.5%), only the basic geometry was displayed, but in a majority of articles (146/156, 93.6%), at least multiple colors or transparencies were used. More advanced features such as PMI/markup (11/156, 7.1%), scripting (29/156, 18.6%), or textures (28/156, 17.9%) were used rarely. Only one article (0.6%) made use of the animation feature [67].

The disciplines were manifold. In most cases either animal (86/200 total article, 43.0%) or human (33/200, 16.5%) anatomies were visualized. The other fields were general science (34/200, 17.0%), biochemistry (14/200, 7.0%), embryology (14/200, 7.0%), clinical/medicine (13/200, 6.5%), biology (3/200, 1.5%), statistics (1/200, 0.5%), bioinformatics (1/200, 0.5%), and astronomy (1/200, 0.5%).

The file size ranged between 0.2 and 429 mebibyte (MiB) (mean 29.9, SD 59.9 MiB). An analysis by year of publication is provided in Table 1; a detailed analysis for each publication is available in Multimedia Appendix 4.

### Three-Dimensional Portable Document Format in Clinical Use Cases

We found 13 publications covering the use of 3D PDF for medical purposes. Eight of them could be assigned to the clinical field, whereas the other five were studies about the value of 3D PDF as educational material for students of medicine. Only one article described a use case from a clinical routine operation: the distribution of results from computer-aided planning for liver surgery [3]. The evaluation of the acceptance and the user experience in this study turned out to be very good. This confirms that the usage of 3D PDF for clinical communication in telemedicine is practicable and highly accepted by the users, and that it has many advantages over 2D technology. The method described there has been used for research as well [221].

**Table 1:** Number of publications dealing with 3D PDF by type (assignment to more than one article type was possible).

Year	Article type, n				Total, n
	Tool	Application	Descriptive	Mentioning	
2008	3	5	—	—	5
2009	—	6	1	—	7
2010	2	9	—	1	13
2011	3	13	1	1	18
2012	2	13	2	1	18
2013	4	20	1	2	24
2014	1	23	5	2	28
2015	1	31	2	—	34
2016	1	18	3	1	25
2017	1	12	2	4	19
2018 (until April)	1	6	—	1	9

All other publications with clinical applications of 3D PDF reported pure research work. Day and colleagues [109] demonstrated that preoperative 3D modeling of magnetic resonance imaging data of fistula-in-ano can reduce confusion and ambiguity among surgeons when interpreting 3D PDF reports instead of complex textual reports. The benefit of 3D PDF for the simulation of surgical procedures was described by Mavar-Haramija and colleagues [4,41] for endoscopic endonasal interventions. Husch and colleagues [192] demonstrated how automatically generated 3D PDF reports can be used to visualize and assess the spatial relationships between electrodes and brain structures and to detect misplaced electrodes in a postoperative deep brain stimulation setting. Finally, the findings of Elbashti and colleagues [54] emphasized the value of 3D PDF for clinical communication with their study on the use of 3D PDF for the documentation of maxillofacial prostheses.

Another area where 3D PDF has proven valuable is patient education. Both the telemedicine study on liver surgery mentioned previously [3] and another study by Prats-Galino and colleagues [197] pointed out that 3D PDF can make it much easier to convey the necessary understanding of a planned procedure, its risks, and future consequences to a patient.

Several other studies showed that 3D PDF is a good means to help students understand anatomy [2,27,42,198] or surgical procedures [199].

### Interim Summary

Overall, within the investigated period of 10 years, only 200 articles were found that used or described the usage of 3D PDFs in biomedical science, with 13 of them covering clinical topics. Compared to the importance of 3D data in the biomedical domain and thus to a presumed number of several thousand publications containing 3D data in some form, this is a tiny fraction. Therefore, we hypothesized about possible reasons for this. We assumed that the lack of 3D PDF usage might be due to one of the following reasons (see Introduction): (1) the existence and/or possibilities of 3D PDF might not be well known among the scientific community, (2) the necessary knowledge that would enable authors to actually make use of 3D PDF might be hard to acquire, and (3) the creation of appropriate model files and of final PDF documents might be an overly cumbersome process.

A verification of these hypotheses would be out of scope of this review. However, assuming these are true, it is possible to mitigate the causes of these hypothesized problems on the basis of the publications that were already collected: hypothesis 1 might be mitigated by the pure existence of this review in general, and both hypotheses 2 and 3 might be mitigated by providing an overview of the protocols and software for creating PDFs that have been published so far.

### Protocols and Software for Creating Three-Dimensional Portable Document Format

Of the total of 200 articles, the tool articles (ie, articles that described a method, protocol, software, library, or other means for the creation of 3D PDFs or model files) accounted for 19 articles (9.5%) [20,40,55-71] (Tables 2 and 3; a combined table is available in Multimedia Appendix 5). These tools can be classified into two groups: (1) protocols that described all necessary artifacts and steps for a creation workflow, and (2) software libraries or software apps that could produce 3D PDFs or necessary intermediate file formats (U3D or PRC).

The following is a description of all these tools (software and protocols) that have been published in the biomedical field. It is intended to be used as a reference for authors that are interested in using 3D PDF technology for their publications or for applied medical use (eg, as demonstrated in [3]). However, especially the older protocols rely on software that is no longer available and that has no successor with the same functionality. For a quick overview, see Tables 2 and 3, and see Multimedia Appendix 5 for a more detailed analysis.

The first protocol that described the workflow for the creation of a 3D PDF was presented by Kumar and colleagues [55] in 2008 and required a minimum of four steps as well as the usage of commercial software and three intermediate file formats. It is based mainly on Adobe Acrobat 3D, which is no longer available today. Another very simple protocol was presented by Barnes and Fluke [222] in 2008: along with their S2PLOT [223] library for the programming languages C, C++, and Fortran, only the Adobe Acrobat 3D software was needed. The S2PLOT library was later extended to provide additional features for creating PRC files (described subsequently). At around the same time, Ruthensteiner and Heß [57] published their protocol for the creation of 3D PDFs of biological specimens. It consisted of 11 steps, including four related to the processing of the sample itself. This protocol relied on commercial software as well: Adobe Acrobat 3D and Adobe 3D Toolkit, both of which are no longer available today.

Approximately two years later, Kumar and colleagues [58] presented a protocol for the generation of 3D PDFs of molecules that required a minimum of four steps. Although this protocol was generally based on commercial software, which in part is no longer available, it also shows an alternative way by using the well-known document preparation system LaTeX [224] in combination with the movie15 package [225]. However, the use of LaTeX requires a model file in U3D format, which needs to be created with other software.

Two months later, Ruthensteiner and colleagues [59] presented an update of the 2008 article that included the S2PLOT library and that focused on virtual volume rendering in PDF documents. This protocol included for the first time the scripting feature of PDF. The need for commercial software, however, had still not been eliminated. At the beginning of 2012, de Boer and colleagues [60] published a very detailed protocol for the creation of 3D PDFs that described all necessary steps for a basic process and an advanced process on seven pages. It also relied on commercial software (Amira, Adobe Acrobat 9 Pro Extended, Adobe Illustrator), but for the first time it allowed for embedding custom control elements for selecting structures of predefined views of the 3D figure. One month later, Ziegler and colleagues [40] demonstrated how to incorporate a variety of multimedia elements into PDF, including a textured 3D figure (an interactive model of a human face). This publication also mentioned the possibility of using LaTeX to create the final PDF. In August 2011, Danz and Katsaros [61] published their protocol, which was based on a combination of free software (for model generation) and commercial software (for PDF creation). One year later, Shin and colleagues [62] also released a protocol that was still purely based on commercial software; 4 months later, Phelps and colleagues [63] presented another solution that combined a workflow of 22 steps using commercial and noncommercial software. Finally, Lautenschlager [64] published in 2013 another protocol that required a wide range of commercial and noncommercial software.

**Table 2:** Overview of requirements, restrictions, and possible output of the tools presented in the tool articles. For features, see Table 3.

Article		Software needed		Output	Programming needed^a^
		Commercial	Free		
**Articles presenting a protocol**					
	Kumar et al [55]	Adobe Acrobat 3D^b^, Adobe Acrobat 3D Toolkit^c^, Adobe Photoshop, Adobe Illustrator		Model file, final PDF	No
	Barnes & Fluke [56]	Adobe Acrobat 3D^b^	S2PLOT	Final PDF	Yes
	Ruthensteiner & Heß [57]	Amira, Adobe Acrobat 3D Toolkit^b^, Adobe Acrobat 3D^b^		Model file, final PDF	No
	Kumar et al [58]	Adobe Acrobat 9 Pro Extended^d^, Adobe Acrobat 3D Reviewer^b^	LaTeX, MeshLab	Model file, final PDF	No
	de Boer et al [60]	Amira, Adobe Acrobat 9 Pro Extended^d^, Adobe Illustrator		Final PDF	No
	Ziegler et al [40]	Adobe Acrobat 9 Pro Extended^c,d^	LaTeX	Final PDF	No
	Danz & Katsaros [61]	Adobe Acrobat X Pro^d^, Tetra 4D 3D PDF Converter plug-in for Acrobat	MeshLab, DAZ Studio		No
	Shin et al [62]	Mimics, Autodesk Maya, Deep Exploration^d^, Adobe Acrobat 9 Pro Extended^d^		Final PDF	No
	Phelps et al [63]	Microsoft PowerPoint, Adobe Acrobat	MeshLab	Model file, final PDF	No
	Lautenschlager [64]	Amira, Mimics, Adobe Acrobat 8 Pro^b^/9 Pro Extended^d^, Adobe Acrobat 3D Reviewer^b^, Adobe Acrobat 3D Toolkit^b^, Tetra 4D 3D PDF Converter plug-in for Acrobat, GeoMagic Studio^c^, Abaqus FEA^c^, Avizo^c^, VG Studio Max^c^, Autodesk Maya^c^	MeshLab, LaTeX, Drishti^d^, SPIERSedit^d^, Blender^d^, DAZ Studio^d^	Model file, final PDF	No
	Mavar-Haramija et al [65]	Amira, Adobe Acrobat 9 Pro Extended^d^, Adobe Acrobat 3D Reviewer^b^, Tetra 4D 3D PDF Converter plug-in for Acrobat		Model file, final PDF	No
	van de Kamp et al [67]	Amira or Avizo, Cinema 4D, Deep Exploration^d^, Adobe Acrobat 9 Pro Extended^d^		Model file, final PDF	No
	Zhang et al [70]		libHaru	Model file, final PDF	Yes
**Articles presenting a protocol and a library**					
	Ruthensteiner et al [59]	Deep Exploration^d^, Adobe Acrobat 9 Pro Extended^d^	Amira, S2PLOT, ImageMagick,	Model file, final PDF	Yes
	Barnes et al [20]		Asymptote, S2PLOT, LaTeX, libHaru	Model file, final PDF	Yes
**Articles presenting a software app**					
	Newe & Ganslandt [66]		MevisLab	Model file	No
	Newe [68]		MevisLab	Model file	No
	Newe [69]		MevisLab	Model file, final PDF	No
	Brandner et al [71]		MevisLab	Model file, final PDF	No

^a^Solution requires the creation of individually tailored software code. Usage of LaTeX is not considered as “programming.”

^b^Software is no longer available.

^c^Optional.

^d^Software is no longer available, but a successor which provides the same functionality is available.

**Table 3:** Overview of features of the tools presented in the tool articles (PDF: Portable Document Format).

Article		Supported geometry type						Supported 3D PDF features											
		Mesh		Polyline		Point cloud		Multiple colors		PMI/markup		Scripting		Texture		Animation		Poster image	
**Articles presenting a protocol**																			
	Kumar et al [55]		x		x		x		x				x		x		x		x
	Barnes & Fluke [56]		x		x		x		x				x		x				
	Ruthensteiner & Heß [57]		x		x		x		x				x		x		x		x
	Kumar et al [58]		x						x				x						
	de Boer et al [60]		x						x		x		x		x				
	Ziegler et al [40]		x		x		x		x		x		x		x				
	Danz & Katsaros [61]		x																
	Shin et al [62]		x						x						x				
	Phelps et al [63]		x						x										
	Lautenschlager [64]		x						x						x		x		x
	Mavar-Haramija et al [65]		x		x		x		x				x						
	van de Kamp et al [67]		x						x								x		
	Zhang et al [70]		x		x		x		x										
**Articles presenting a protocol and a library**																
	Ruthensteiner et al [59]		x		x		x		x		x		x		x				x
	Barnes et al [20]		x		x		x		x		x		x		x		x		
**Articles presenting a software app**																			
	Newe & Ganslandt [66]		x						x										
	Newe [68]		x		x		x		x										
	Newe [69]		x		x		x		x										x
	Brandner et al [71]		x		x		x		x						x				x

The first solution that could be used without any commercial software was presented by Barnes and colleagues [20] in September 2013 (ie, 5 years after the first appearance of 3D PDF in biomedical publications). It was based on a number of free software tools and libraries (Asymptote [226]; S2PLOT; LaTeX, libHaru [227]). Although this solution could be used to create both model files and final PDFs, and it could be used free of charge, it had a major drawback: it required writing a program (ie, a certain set of programming skills was necessary to apply this protocol). On the other hand, this gave the user greater flexibility because the program code could be adapted exactly to their needs and to the specific-use case.

Only 2 months later, two more articles with tools for the creation of 3D PDFs were published. Mavar-Haramija and colleagues [4] presented another commercial software-based protocol that focused on control elements for a more sophisticated interaction with the 3D model. At the same time, Newe and Ganslandt [66] presented a noncommercial solution for the creation of model files of surface meshes in U3D format that did not require programming skills. The final PDF could not be created with that solution, but it reduced the number of steps for the creation of such model files and provided a convenient graphical user interface. However, this solution required the installation of the third-party biomedical-imaging framework, MeVisLab [228,229].

In September 2014, a previously unregarded feature of 3D PDF emerged through a new protocol: animation. Van de Kamp and colleagues [67] for the first time demonstrated the feasibility and the usefulness of animated 3D figures in electronic publications. Eight months later, Newe [68] presented an update to his previous publication that provided an advanced user interface for more flexibility. Furthermore, it provided the possibility to embed both point clouds and line sets into the interactive 3D figures. Previous publications usually only considered objects that consisted of surface meshes. However, it was still limited to the creation of model files. The creation of final PDFs was not possible.

The first all-in-one solution for the creation of both model files and the final PDF that did not require any programming was also presented by Newe [69]. The Scientific3DFigurePDFApp provided a graphical user interface for the assembly of 3D models (meshes, line set, point clouds) and included an editor for predefined views. It was also capable of producing one-paged PDF files with the embedded 3D figure that could directly be used as supplementary figures for scientific publications.

The latest complete protocol was published by Zhang and colleagues [70] in November 2017. Although it did not require commercial software, it was also based on the libHaru library (ie, it required the writing of program code). However, this publication featured a deep insight into the U3D file format.

Finally, Brandner and colleagues [71] presented an extension to the toolbox described in [69], which added the support of textures.

## Discussion

### Summary of Main Findings

With this systematic review, we give an overview of the usage of 3D PDF in the biomedical domain with a special focus on clinical applications. Furthermore, we investigate which protocols and software tools for 3D PDF creation have been published so far. In addition, we hypothesize about possible barriers that might be responsible for the low dissemination of 3D PDF in clinical communication and biomedical sciences. Our primary search revealed 19 tool articles that can be considered as the roots of 3D PDF in biomedical publishing. For these articles, the scope has intentionally not been limited to the biomedical domain, because many biomedical publications seem to be inspired by the Barnes [56] publication (ie, an astronomy publication). Excluding this paper would have left several in-scope biomedical articles unrevealed (eg, [73,88,121,128,131]).

After the secondary search, in which we searched for articles that cited these tool articles, 200 articles were found overall that fulfilled the inclusion criteria and entered into further analysis. This is a very small number with respect to the fact that in the last 10 years (January 1, 2008 to January 1, 2018) a total number of 9,705,959 articles were indexed by PubMed. Even if only 1% of these articles touched the subject of 3D data, 200 articles is still a tiny fraction.

Within these 200 articles, actual 3D PDF figures were available in 156 articles (78.0%). However, the 3D content was embedded directly into the PDF version of the articles in 34 cases only. One reason for this might have been that the journals did not support the direct embedding. This matches the experience of the authors. Even some modern, online-only journals are not capable (and, in some cases, not willing) to provide the possibility to embed 3D figures directly into the PDF versions of their articles.

### Possible Reasons for the Low Dissemination of Three-Dimensional Portable Document Format in Medicine and Biomedical Research

As mentioned previously, one reason might be that the journals do not support 3D PDFs. However, a further—and probably more serious reason—might be that most clinicians and scientists do not know about this feature. Although this hypothesis could not be investigated with our systematic review and it should be investigated in future research, the pure existence of this review article might help to draw attention to this technology and help to overcome this threshold.

A further reason might be that the potential target audience—although they know about 3D PDF in principle—do not know how to create 3D PDFs or that they do not have appropriate technical skills. In our systematic review, we reported the protocols and software tools that were used in the 156 articles that apply 3D technology in PDFs. Unfortunately, many of them are based on software packages that are no longer available. Furthermore, the usage of most of the reported protocols or tools needed either programming skills, advanced technical knowledge, and/or expensive, highly specialized, commercial software. Although this gives the user great flexibility because the program code can be adapted exactly to the needs and to the specific-use case, we conclude that the threshold for creating 3D PDFs is still too high and that easy-to-use, standalone software tools are needed to facilitate 3D PDF creation. Of all the software tools analyzed in this review, the solution presented by Newe [69] comes closest to this requirement because it is an all-in-one solution for the creation of both model files and final PDFs, which is operated via a graphical user interface. The major drawback of this solution is the need for the software MeVisLab, which also requires some basic training.

### Potential of Three-Dimensional Portable Document Format and Future Research

Although it is not widely disseminated yet, the first approaches are very promising and show that 3D PDF has a high potential in clinical communication, in biomedical research, and in research in general. 3D PDF offers the possibility to simply distribute atlases of, for example, human or animal anatomy as demonstrated in the 3D Atlas of Human Embryology [110,200,230] or in the Visible Korean anatomy database [62,231]. It is, therefore, an alternative to traditional 2D atlases in which much of information gets lost because of the projection of a 3D object to a 2D plane [18]. Furthermore, 3D PDFs can easily be distributed via the internet or shared via emails, making it unnecessary to travel to museums all around the world, for example.

Future research should mainly focus on the development of software tools that can be used easily by everyone and without charge. Furthermore, it should be investigated (eg, by means of surveys) how well-known 3D PDF is in the biomedical and clinical community. Besides, simple instruction manuals for the existing tools are needed and should be disseminated further. Additionally, the routine usage of 3D PDF in clinical applications should be further promoted and evaluated for other disciplines than liver surgery planning. Finally, future research could also consider other domains than the biomedical one.

### Limitations

Our review is subject to some limitations. First, an unknown number of articles that would fulfill the inclusion criteria may not have been found. The most probable reason for missing such an article might be that authors developed the 3D PDF on their own (without any of the tool articles) and that the availability of a 3D PDF was not necessarily pointed out in the abstract or in the available full text. Another reason might be that a tool article was used, but not properly cited. However, 200 articles can be considered a solid basis for a review.

Based on our expertise and the high relevance of 3D PDFs in the investigated domain, the scope of this review was intentionally limited to biomedical publications. Other scientific disciplines are known to use 3D PDF as well (eg, astronomy [232], paleontology [233,234], and chemistry [235]) and may yield different results.

Second, as already mentioned, many of the protocols presented previously are based on commercial software that is no longer available and that has no successor providing the same functionality. To cover all available articles, they are still presented, but the use of the more modern protocols is recommended.

Third, a systematic review is not a suitable method to investigate the personal reasons for researchers not using 3D PDF. Other methods such as surveys are needed for this.

### Conclusions

The use of 3D PDF for visualization purposes in real medical use cases or in biomedical publications is not yet fully accepted, although the necessary technique is available and there are many arguments in favor of 3D PDF. In this review paper, a wide range of examples for applied 3D PDF technology and a variety of protocols and software tools for the creation of relevant documents and files are presented. It aims to draw attention to this valuable technology, to demonstrate the possibilities, to help interested readers find a suitable solution, and lower the threshold for its use.

## Appendix

Multimedia Appendix 1 3D PDF version of Figure 1.

Multimedia Appendix 2 3D PDF version of this article (best viewed with Adobe Reader 9 or later).

Multimedia Appendix 3 Overview over the search strategy and all articles that were found and processed.

Multimedia Appendix 4 Detailed analysis of all eligible articles.

Multimedia Appendix 5 Detailed analysis of articles that present a protocol or a software for 3D PDF creation.

## Abbreviations

2D: two-dimensional
3D: three-dimensional
ECMA: European Computer Manufacturers Association
ISO: International Organization for Standardization
MiB: mebibyte, (binary) megabytes
PDF: Portable Document Format
PMI: product manufacturing information
PRC: Product Representation Compact
U3D: Universal 3D
