# Supplementary material for: Three-Dimensional Portable Document Format (3D PDF) in Clinical Communication and Biomedical Sciences: Systematic Review of Applications, Tools, and Protocols
Source: JMIR Med Inform. 2018 Aug 7;6(3):e10295. doi: 10.2196/10295 (PMC6103636; doi:10.2196/10295)
Supplement: Multimedia Appendix 1 [file medinform_v6i3e10295_app1.pdf]

Newe & Becker (2018) *Three-Dimensional Portable Document Format (3D PDF) in Clinical Communication and Biomedical Sciences: Systematic Review of Applications, Tools, and Protocols*. JMIR Med Inform.
doi: 10.2196/10295.

Multimedia Appendix 1 - 3D PDF of Figure 1

Figure is best viewed with Adobe Reader 9 or later

Click inside image or frame to enable interactive mode.
- Left-click & move mouse to rotate scene.
- Right-click & move mouse to zoom.
- Both-click and move mouse to pan.

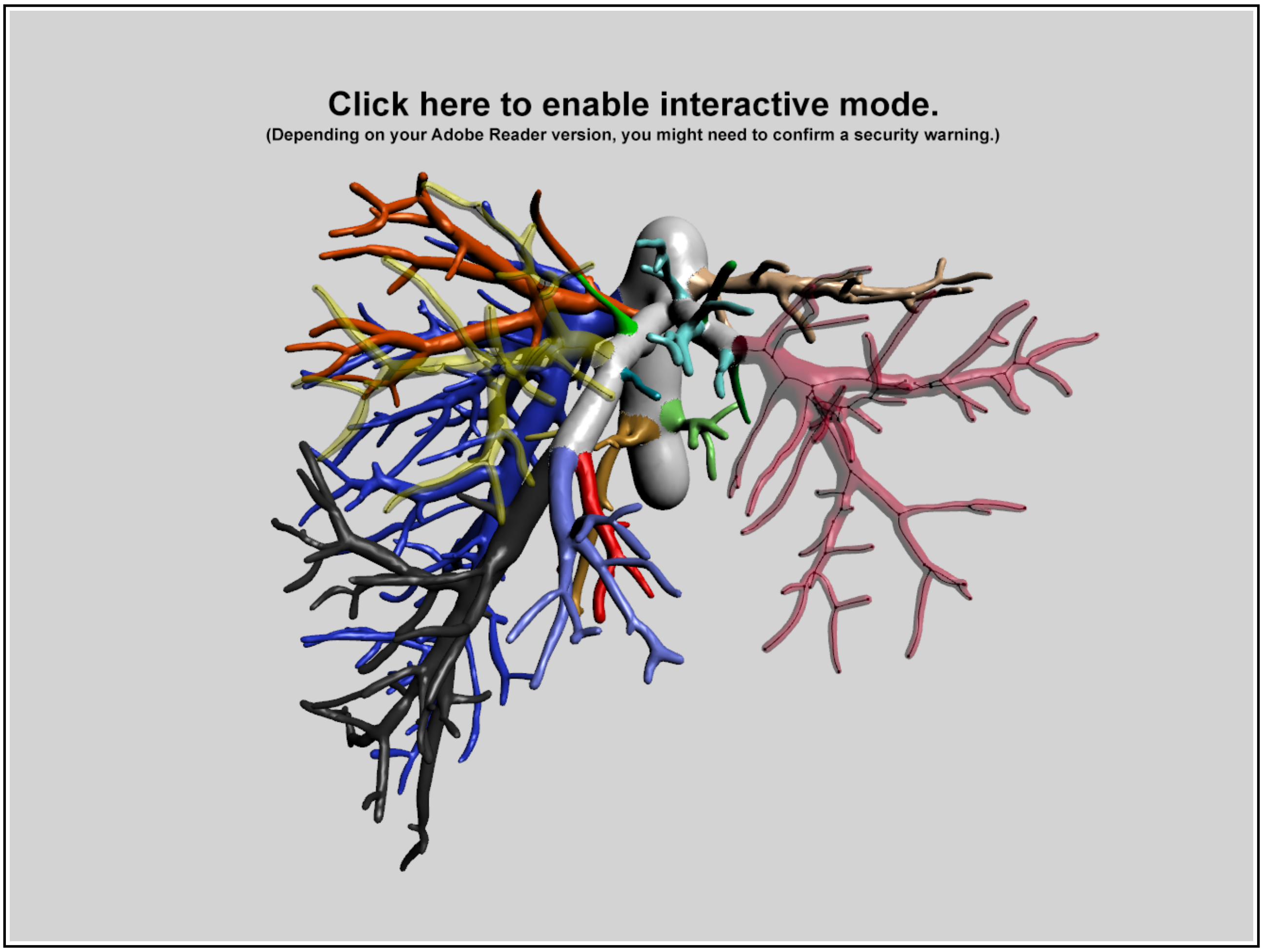

Example for an interactive 3D scene in PDF depicting the vessel tree of a liver.

Click into the figure to enable interactive mode in the PDF version of this article (Adobe Reader 9 or later or another 3D-capable PDF reader is required). Left-click and move mouse to rotate scene. Right-click and move mouse to zoom. Both-click and move mouse to pan.

This page was created by means of the software presented in Newe (2016) Enriching scientific publications with interactive 3D PDF: an integrated toolbox for creating ready-to-publish figures. PeerJ Computer Science 2:e64. doi: 10.7717/peerj-cs.64.
