# Supplementary material for: Three-Dimensional Portable Document Format (3D PDF) in Clinical Communication and Biomedical Sciences: Systematic Review of Applications, Tools, and Protocols
Source: JMIR Med Inform. 2018 Aug 7;6(3):e10295. doi: 10.2196/10295 (PMC6103636; doi:10.2196/10295)
Supplement: Multimedia Appendix 2 [file medinform_v6i3e10295_app2.pdf]

Review

Three-Dimensional Portable Document Format (3D PDF) in Clinical Communication and Biomedical Sciences: Systematic Review of Applications, Tools, and Protocols

Axel Newe[1,2], Dipl-Ing (FH), Dr; Linda Becker[3], Dipl-Psych, Dipl-Phys, Dr

[1]Chair of Medical Informatics, Friedrich-Alexander University Erlangen-Nürnberg, Erlangen, Germany
[2]NewTec GmbH, Pfaffenhofen an der Roth, Germany
[3]Chair of Health Psychology, Friedrich-Alexander University Erlangen-Nürnberg, Erlangen, Germany

Corresponding Author:
Axel Newe, Dipl-Ing (FH), Dr
Chair of Medical Informatics
Friedrich-Alexander University Erlangen-Nürnberg
Wetterkreuz 13
Erlangen, 91058
Germany
Phone: 49 913185 ext 26720
Fax: 49 91318526754
Email: axel.newe@fau.de

Abstract

Background: The Portable Document Format (PDF) is the standard file format for the communication of biomedical information via the internet and for electronic scholarly publishing. Although PDF allows for the embedding of three-dimensional (3D) objects and although this technology has great potential for the communication of such data, it is not broadly used by the scientific community or by clinicians.

Objective: The objective of this review was to provide an overview of existing publications that apply 3D PDF technology and the protocols and tools for the creation of model files and 3D PDFs for scholarly purposes to demonstrate the possibilities and the ways to use this technology.

Methods: A systematic literature review was performed using PubMed and Google Scholar. Articles searched for were in English, peer-reviewed with biomedical reference, published since 2005 in a journal or presented at a conference or scientific meeting. Ineligible articles were removed after screening. The found literature was categorized into articles that (1) applied 3D PDF for visualization, (2) showed ways to use 3D PDF, and (3) provided tools or protocols for the creation of 3D PDFs or necessary models. Finally, the latter category was analyzed in detail to provide an overview of the state of the art.

Results: The search retrieved a total of 902 items. Screening identified 200 in-scope publications, 13 covering the use of 3D PDF for medical purposes. Only one article described a clinical routine use case; all others were pure research articles. The disciplines that were covered beside medicine were many. In most cases, either animal or human anatomies were visualized. A method, protocol, software, library, or other tool for the creation of 3D PDFs or model files was described in 19 articles. Most of these tools required advanced programming skills and/or the installation of further software packages. Only one software application presented an all-in-one solution with a graphical user interface.

Conclusions: The use of 3D PDF for visualization purposes in clinical communication and in biomedical publications is still not in common use, although both the necessary technique and suitable tools are available, and there are many arguments in favor of this technique. The potential of 3D PDF usage should be disseminated in the clinical and biomedical community. Furthermore, easy-to-use, standalone, and free-of-charge software tools for the creation of 3D PDFs should be developed.

(JMIR Med Inform 2018;6(3):e10295) doi:10.2196/10295

KEYWORDS

3D PDF; 3D visualization; interactive; clinical communication; biomedical science; tools; protocols; apps; online data sharing

XSL•FO
RenderX

Introduction

Background

The best-known and most widely used data format for the exchange of electronic documents is probably the Portable Document Format (PDF), which was standardized by the International Organization for Standardization (ISO) as ISO 32000-2:2017 [1]. Software that can read PDFs is installed on nearly every computer, and most internet browsers and email clients have a built-in PDF renderer. This, in many cases, makes this format the best means for the exchange of electronic documents. However, the PDF offers more features than many people are aware of. Although technically available since 2005, a still lesser-known standard feature of the PDF is the possibility to embed three-dimensional (3D) models, which enables the interactive visualization (eg, zooming, panning, rotating, and selection of components) of such objects with qualified reader software (Figure 1 and Multimedia Appendix 1).

A PDF document with embedded 3D objects (3D PDF) has high potential in almost every scenario in which 3D objects should be visualized and exchanged between different platforms (eg, computers with different operating systems). A highly relevant use case is the exchange of medical and biomedical data (eg, the visualization of human anatomy for medical students [2] or clinical data such as the results of surgery planning [3,4]). Therefore, this feature is also considered in the standard AIIM/ASTM BP-01-2008 "Portable Document Format-Healthcare (PDF) A Best Practices Guide" (also known as PDF Healthcare or PDF/H) [5,6]. This standard describes how to use the PDF to exchange and preserve digital health care information in a safe and secure way.

In addition to the exchange between a few persons (eg, a doctor and a patient), 3D PDF offers a very efficient and convenient way for distributing 3D structures through the scientific community by embedding 3D objects into scientific publications.

The primary aim of this review was to investigate the significance of 3D PDF in clinical communication and in scholarly publications in the biomedical sciences. The secondary aim of this review was to provide an overview of the technical possibilities and to present all currently available solutions for creating 3D PDFs and related model files.

The Portable Document Format

The PDF is a computer file format for the platform-independent definition of electronic documents [1]. It allows for describing electronic documents with preserved fidelity, independently of the software, device, and operating system used to create, display, or print them. Furthermore, PDF is capable of encapsulating all necessary resources (eg, texts, images, or multimedia elements). Along with the open accessibility of the specification, this has led to PDF being the most commonly used file format for the exchange of electronic documents. For example, all scholarly journals allow for an electronic publication of their articles in PDF today.

The PDF was originally developed by Adobe Systems and evolved in the early 1990s from the PostScript page description language for printers. The first specification of PDF was published in 1993; since then, it has been developed further by Adobe until version 1.7 [7], which was released in 2006. This version was given to the ISO, which re-released this specification as ISO standard 32000-1:2008 in July 2008 [8]. The latest version (PDF 2.0) was published in July 2017 [1,9].

The PDF is now the de facto standard for the exchange of electronic documents. The original Adobe Reader software alone has been distributed more than 500 million times around the world [7], and countless other apps for displaying PDF documents are available. For example, Quartz 2D, the native graphics rendering interface for two-dimensional (2D) graphics of the macOS X and iOS operating systems, is based on the PDF specification, (ie, it is an integral part of these operating systems) [10].

Figure 1. Vessel tree of a liver (interactive 3D figure). See Multimedia Appendix 1 for independent figure.

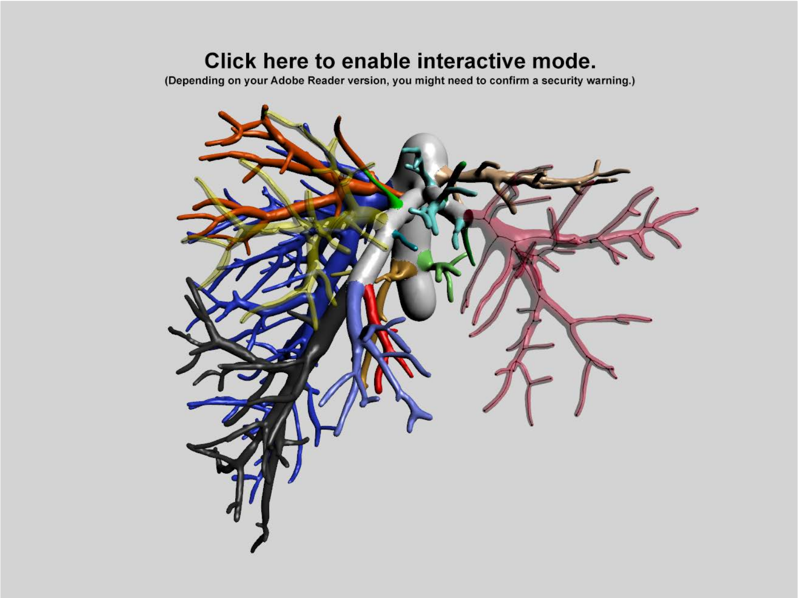

XSL•FO
RenderX

The Development of the Three-Dimensional Portable Document Format

Version 1.6 of the PDF specification, published in 2004 [11], introduced the capability to embed 3D objects, such as those used by software for computer-aided design, into PDF files. At the time of the introduction of this new feature, the format for the data of 3D objects needed to be Universal 3D (U3D), a file format developed by the 3D Industry Forum (which Adobe was part of) as "a single, common, and open 3D standard" and "JPEG for 3D" [12]. U3D is standardized by the European Computer Manufacturers Association (ECMA) as ECMA-363 [13]. The first software to support 3D Artwork (the PDF term for 3D objects in PDF files) was the Adobe Reader 7 (and Adobe Acrobat 7, respectively), published in January 2005. However, the first software that really allowed for an efficient and convenient working with 3D models in PDF was Acrobat 3D, released in early 2006. This version provided tools for the import and conversion of many 3D formats and a 3D editor [14].

In late 2006, Adobe added the ability to embed 3D models in Product Representation Compact (PRC) format into PDF (PDF BaseVersion 1.7, ExtensionLevel 1 [15]), but this feature has not been integrated into the ISO standard 32000-1 from 2008. It took 11 years until the release of PDF 2.0 (32000-2:2017) in July 2017 to formally integrate PRC as 3D format into the PDF standard. However, the support of editing features for 3D was discontinued in later versions of Adobe Acrobat. Acrobat 9 Pro Extended was the last version to include tools for conversion and editing of 3D models. Instead, this functionality has been outsourced to third-party software and has been sold separately since 2010 [16].

Another feature that is notable in this context is the support of JavaScript. This feature was introduced with PDF 1.3 in July 2000 [17] and allows for executing JavaScript commands within a PDF document (eg, after clicking an embedded button or after activating a 3D model).

Relevance of Three-Dimensional Portable Document Format

Especially in the biomedical domain, the importance of 3D data has grown in recent years (eg, visualization of chemical molecules, anatomy, or vessel systems). With the availability of the necessary technology for the visualization of such 3D data, this technology should consequently be used to avoid a loss of information [18]. This is of particular relevance in scholarly publishing [19]. Furthermore, 3D PDF has a very high potential for the exchange of medical data in clinical communication [3] because it allows for in situ publishing of 3D figures [20].

Objectives

It has tremendously high potential for clinical communication, many scholarly articles would benefit from interactive visualization of 3D data [19], publishers encourage their authors to use 3D PDF technology [21,22], and some journals provide the necessary tools [23], yet it still does not seem to be common use. Thus, the initial motivation for writing this review paper was to test this hypothesis (ie, that using 3D PDF technology is not common in clinical communication or in biomedical publications). Therefore, as the primary goal, an overview was to be created of the dissemination of 3D PDFs in biomedical publications. The scope was not limited to purely clinical use cases to cover as wide a range of possible apps in all fields of medically relevant research. Furthermore, we will investigate in which research areas and use cases 3D PDF has been applied since the advent of this technology.

After the initial hypothesis was confirmed (200 articles were found over a period of 10 years; see Results), we hypothesized three possible reasons:

1. The existence and/or possibilities of 3D PDF might not be well known among the scientific and clinical community.
2. The necessary knowledge that would enable authors and possible users to actually make use of 3D PDFs might be hard to acquire.
3. The creation of appropriate model files and of final PDF documents might be an overly cumbersome process.

Therefore, the secondary goal of this review was to tackle the previous three issues by providing an overview of (1) existing publications that apply 3D PDF technology to demonstrate its possibilities, (2) available protocols for the creation of 3D PDFs to provide the basic knowledge in compressed form, and (3) existing software tools for the creation of model files and 3D PDFs to show ways to simplify the process.

Although the overview of the existing publications that apply 3D PDF technology in this field may elaborate new ideas of how this form of visualization could augment scholarly communication in biomedicine, the presented protocols and tools can probably be applied in other fields as well.

Methods

General Procedure

A systematic literature search was performed in the scientific databases PubMed [24] and Google Scholar [25] to find articles that either applied 3D PDF technology for visualization purposes or articles that generally dealt with the subject of 3D PDF in the context of biomedical sciences.

Because PDF is a vehicle for presenting data rather than an actual research topic, it was likely that a simple database search for this term would not reveal comprehensive coverage of the literature, especially for articles that simply used 3D PDF as a means for visualization. Therefore, a search strategy with two iterations was conceived (see detailed description subsequently). Agreement about this strategy, the inclusion criteria, and the review protocol (see respective sections subsequently) was reached through discussion between all authors.

The first search was performed by AN on March 6, 2017. Three update searches were performed by AN on September 30, 2017, January 1, 2018, and April 30, 2018. The search results were screened and checked for eligibility independently by AN and LB on the basis of predefined inclusion criteria. The remaining articles that were entered into the further analysis were reviewed by means of a predefined review protocol by AN and LB independently.

XSL•FO
RenderX

Search Strategy

As mentioned previously, we conceived a two-tiered search strategy (Figure 2). The first iteration was a PubMed search with the following search term: "(((3d) OR (3-d) OR (three dimensional) OR (interactive) OR (surface model)) AND ((pdf) OR (portable document format))) AND ("2005"[Date-Publication]: "3000"[Date-Publication])".

The goal of this initial search was to reveal all biomedical publications that mentioned 3D PDFs without the most common unwanted meanings for "PDF." It was limited to articles published after 2004 because 3D PDF technology was not available before 2005.

The results of this first iteration were then screened to exclude articles that did not fulfill the inclusion criteria. The remainders, which will subsequently be referred to as "primary results," were then analyzed to identify articles that were cited as a source of 3D PDF creation (referred to as "tool articles;" see a more detailed explanation subsequently). Next, both PubMed and Google Scholar were searched for articles that cited these tool articles to identify more articles that applied or mentioned 3D PDF and that could not be found via the initial PubMed search. The results of this second iteration were then also screened for the inclusion criteria and the remaining articles (referred to as "secondary results") went into the review process along with the primary results and the tool articles.

Finally, a small number of additional articles that were known to us to be eligible, but which were not discovered by the systematic search, were included as well. Further details about the search strategy, the search results, and the screening results are available in Multimedia Appendix 3.

Inclusion Criteria

The general inclusion criteria for this review were defined as follows: (1) articles from the biomedical domain that applied 3D PDF for visualization or articles that presented protocols or tools for the creation of 3D PDFs or for the creation of 3D PDF-specific, intermediate file formats (U3D or PRC); (2) articles in English language only; and (3) peer-reviewed articles only.

Review Protocol

To fulfill all these objectives, all eligible articles were first classified into article types using four categories:

1. Application: articles that applied 3D PDF technology for visualization.
2. Descriptive: articles that described a way, method, or idea how to use or to utilize 3D PDF technology.
3. Tool: articles that described a method, protocol, software, library, or other means for the creation of 3D PDFs or model files.
4. Mentioning only: articles that only mentioned the possibility of using 3D PDF (without further details).

The assignment to more than one category (eg, descriptive and application) was allowed.

Figure 2. Search strategy.

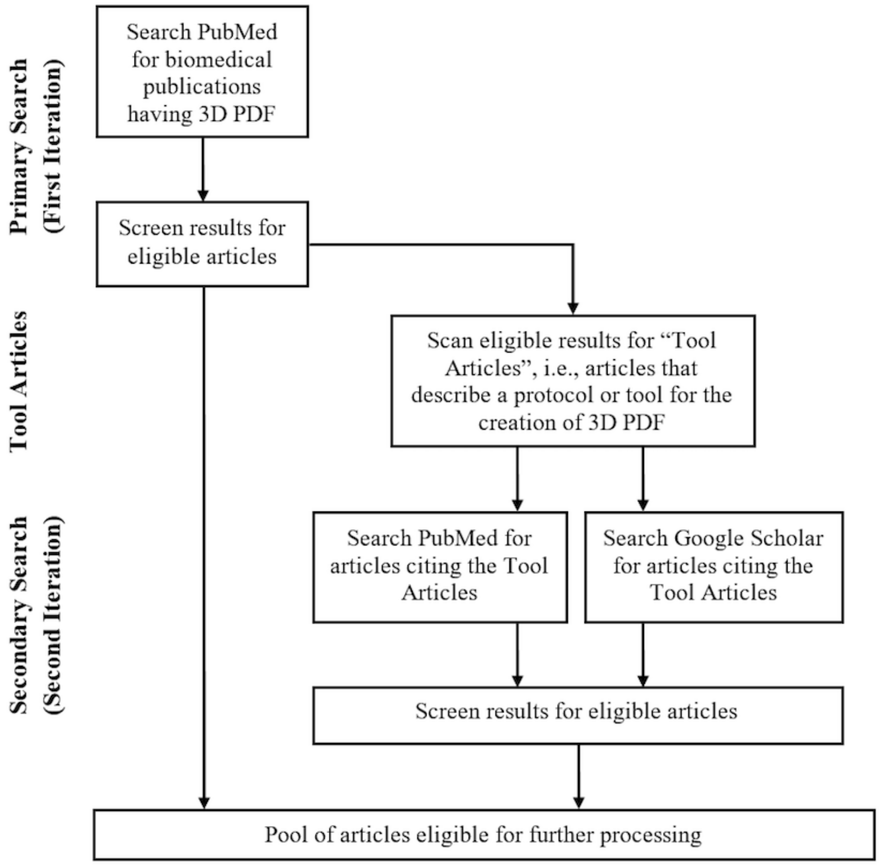

XSL•FO
RenderX

All articles of the type “application” were then further analyzed for the 3D features used, for the availability of the 3D PDF documents, and for the file size. The following 3D PDF features were considered:

1. Geometry only: articles that only presented the geometry of the 3D model without further features.
2. Multiple colors and transparency: articles that had transparent/colored models.
3. Product Manufacturing Information (PMI)/markup: articles that used PMI features or markup for labeling objects.
4. Scripting: articles that used JavaScript for advanced interactive features.
5. Texture: articles that used textured models.
6. Animation: articles that use animations.

The availability of the 3D PDFs was classified into:

1. Embedded: articles that directly embedded 3D models into the PDF version.
2. Supplement: articles that provided supplemental information files with 3D PDFs.
3. Link to external resources: articles that provided the URL or mail address for accessing the referred 3D PDFs.

Finally, all articles were classified into a scientific discipline (eg, “medicine,” “embryology,” or “human anatomy”; for a full list see Multimedia Appendix 4) and the clinically relevant articles were analyzed further.

Results

Search Results

The initial PubMed search retrieved 123 publications; after the third update search, a total of 237 publications were found. After screening these PubMed results, 109 articles were excluded because “PDF” was not used as an acronym for “Portable Document Format,” one was excluded because it was not in English language, one was excluded because it did not cover a biomedical topic, and eight were excluded because they were an erratum only (ie, not a peer-reviewed article). During the full-text eligibility assessment, 65 more articles were excluded because they had no content relevant for 3D PDF. The initial analysis of the remaining 53 primary results revealed 19 tool articles, with 15 of them being duplicates (ie, four new tool articles were identified).

The second iteration (ie, the search for articles citing the tool articles) retrieved a total of 128 articles from PubMed and 537 articles from Google Scholar after the third update. After removing 356 duplicates, 366 articles were screened. Of those, 22 were excluded because they were not in the English language and 43 were excluded because they were not peer reviewed. During the full-text eligibility assessment, 48 articles were excluded because they did not fall into the biomedical domain and 63 more articles were excluded because they had no content relevant for 3D PDF. That resulted in 133 articles from the second iteration. Together with the 53 articles of the first iteration, the four additional tool articles, and 10 articles that were included due to our knowledge, 200 articles were included in the final analysis, which is shown in a modified Preferred Reporting Items for Systematic Reviews and Meta-Analyses (PRISMA) flow diagram [26] in Figure 3.

Overall Analysis Results

Of the total 200 articles, the simple possibility of using 3D PDF (without providing further details) was mentioned in 13 articles (6.5%) [27-39]. Another 18 (9.0%) [2,3,23,40-54] articles described a way, method, or idea how to use or to utilize 3D PDF technology and 19 articles (9.5%) [20,40,55-71] described a method, protocol, software, library, or other means for the creation of 3D PDFs or model files.

In 156 (78.0%) articles, 3D PDF technology was actually applied for the visualization of research results. In 11 (7.1%) more articles, it was claimed that 3D PDF should be available, but no 3D model could be found [58,72-81]. Of the 156 articles with applied 3D PDF technology, 34 (21.8%) [40,55-57,61,64,68,82-108] had the 3D content directly embedded into the PDF of the article, 94 (60.3%) [20,48,59,60,63,66,67,109-195] articles provided the 3D content as supplementary material, and 28 (17.9%) [4,41,62,196-220] referred to an external resource. The 34 articles in which the 3D objects were embedded directly into PDF of the main article were published in 25 different journals.

Different features of 3D PDF were used. In seven articles (4.5%), only the basic geometry was displayed, but in a majority of articles (146/156, 93.6%), at least multiple colors or transparencies were used. More advanced features such as PMI/markup (11/156, 7.1%), scripting (29/156, 18.6%), or textures (28/156, 17.9%) were used rarely. Only one article (0.6%) made use of the animation feature [67].

The disciplines were manifold. In most cases either animal (86/200 total article, 43.0%) or human (33/200, 16.5%) anatomies were visualized. The other fields were general science (34/200, 17.0%), biochemistry (14/200, 7.0%), embryology (14/200, 7.0%), clinical/medicine (13/200, 6.5%), biology (3/200, 1.5%), statistics (1/200, 0.5%), bioinformatics (1/200, 0.5%), and astronomy (1/200, 0.5%).

The file size ranged between 0.2 and 429 mebibyte (MiB) (mean 29.9, SD 59.9 MiB). An analysis by year of publication is provided in Table 1; a detailed analysis for each publication is available in Multimedia Appendix 4.

Three-Dimensional Portable Document Format in Clinical Use Cases

We found 13 publications covering the use of 3D PDF for medical purposes. Eight of them could be assigned to the clinical field, whereas the other five were studies about the value of 3D PDF as educational material for students of medicine. Only one article described a use case from a clinical routine operation: the distribution of results from computer-aided planning for liver surgery [3]. The evaluation of the acceptance and the user experience in this study turned out to be very good. This confirms that the usage of 3D PDF for clinical communication in telemedicine is practicable and highly accepted by the users, and that it has many advantages over 2D technology. The method described there has been used for research as well [221].

XSL•FO
RenderX

Figure 3. Modified PRISMA flowchart.

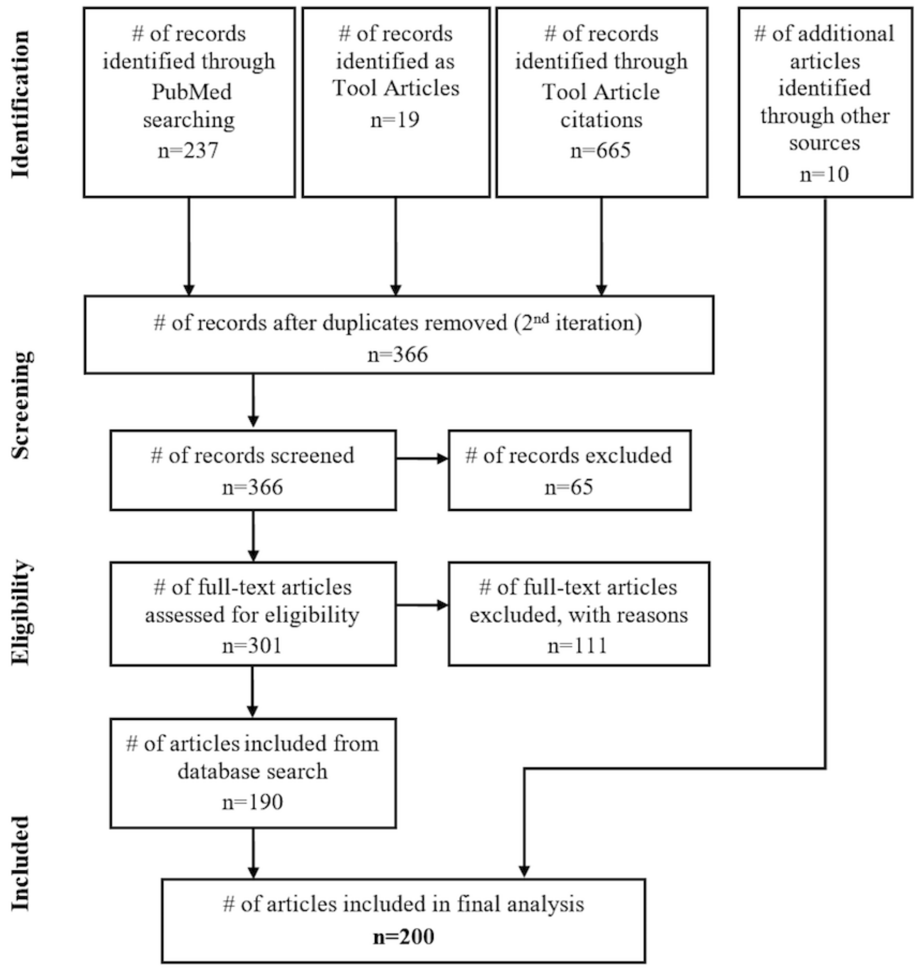

Table 1. Number of publications dealing with 3D PDF by type (assignment to more than one article type was possible).

Year	Article type, n				Total, n
	Tool	Application	Descriptive	Mentioning	
2008	3	5	—	—	5
2009	—	6	1	—	7
2010	2	9	—	1	13
2011	3	13	1	1	18
2012	2	13	2	1	18
2013	4	20	1	2	24
2014	1	23	5	2	28
2015	1	31	2	—	34
2016	1	18	3	1	25
2017	1	12	2	4	19
2018 (until April)	1	6	—	1	9

All other publications with clinical applications of 3D PDF reported pure research work. Day and colleagues [109] demonstrated that preoperative 3D modeling of magnetic resonance imaging data of fistula-in-ano can reduce confusion and ambiguity among surgeons when interpreting 3D PDF reports instead of complex textual reports. The benefit of 3D PDF for the simulation of surgical procedures was described by Mavar-Haramija and colleagues [4,41] for endoscopic endonasal interventions. Husch and colleagues [192] demonstrated how automatically generated 3D PDF reports can be used to visualize and assess the spatial relationships between electrodes and brain structures and to detect misplaced electrodes in a postoperative deep brain stimulation setting. Finally, the findings of Elbashti and colleagues [54] emphasized the value

XSL•FO
RenderX

of 3D PDF for clinical communication with their study on the use of 3D PDF for the documentation of maxillofacial prostheses.

Another area where 3D PDF has proven valuable is patient education. Both the telemedicine study on liver surgery mentioned previously [3] and another study by Prats-Galino and colleagues [197] pointed out that 3D PDF can make it much easier to convey the necessary understanding of a planned procedure, its risks, and future consequences to a patient.

Several other studies showed that 3D PDF is a good means to help students understand anatomy [2,27,42,198] or surgical procedures [199].

Interim Summary

Overall, within the investigated period of 10 years, only 200 articles were found that used or described the usage of 3D PDFs in biomedical science, with 13 of them covering clinical topics. Compared to the importance of 3D data in the biomedical domain and thus to a presumed number of several thousand publications containing 3D data in some form, this is a tiny fraction. Therefore, we hypothesized about possible reasons for this. We assumed that the lack of 3D PDF usage might be due to one of the following reasons (see Introduction): (1) the existence and/or possibilities of 3D PDF might not be well known among the scientific community, (2) the necessary knowledge that would enable authors to actually make use of 3D PDF might be hard to acquire, and (3) the creation of appropriate model files and of final PDF documents might be an overly cumbersome process.

A verification of these hypotheses would be out of scope of this review. However, assuming these are true, it is possible to mitigate the causes of these hypothesized problems on the basis of the publications that were already collected: hypothesis 1 might be mitigated by the pure existence of this review in general, and both hypotheses 2 and 3 might be mitigated by providing an overview of the protocols and software for creating PDFs that have been published so far.

Protocols and Software for Creating Three-Dimensional Portable Document Format

Of the total of 200 articles, the tool articles (ie, articles that described a method, protocol, software, library, or other means for the creation of 3D PDFs or model files) accounted for 19 articles (9.5%) [20,40,55-71] (Tables 2 and 3; a combined table is available in Multimedia Appendix 5). These tools can be classified into two groups: (1) protocols that described all necessary artifacts and steps for a creation workflow, and (2) software libraries or software apps that could produce 3D PDFs or necessary intermediate file formats (U3D or PRC).

The following is a description of all these tools (software and protocols) that have been published in the biomedical field. It is intended to be used as a reference for authors that are interested in using 3D PDF technology for their publications or for applied medical use (eg, as demonstrated in [3]). However, especially the older protocols rely on software that is no longer available and that has no successor with the same functionality. For a quick overview, see Tables 2 and 3, and see Multimedia Appendix 5 for a more detailed analysis.

The first protocol that described the workflow for the creation of a 3D PDF was presented by Kumar and colleagues [55] in 2008 and required a minimum of four steps as well as the usage of commercial software and three intermediate file formats. It is based mainly on Adobe Acrobat 3D, which is no longer available today. Another very simple protocol was presented by Barnes and Fluke [222] in 2008: along with their S2PLOT [223] library for the programming languages C, C++, and Fortran, only the Adobe Acrobat 3D software was needed. The S2PLOT library was later extended to provide additional features for creating PRC files (described subsequently). At around the same time, Ruthensteiner and Heß [57] published their protocol for the creation of 3D PDFs of biological specimens. It consisted of 11 steps, including four related to the processing of the sample itself. This protocol relied on commercial software as well: Adobe Acrobat 3D and Adobe 3D Toolkit, both of which are no longer available today.

Approximately two years later, Kumar and colleagues [58] presented a protocol for the generation of 3D PDFs of molecules that required a minimum of four steps. Although this protocol was generally based on commercial software, which in part is no longer available, it also shows an alternative way by using the well-known document preparation system LaTeX [224] in combination with the movie15 package [225]. However, the use of LaTeX requires a model file in U3D format, which needs to be created with other software.

Two months later, Ruthensteiner and colleagues [59] presented an update of the 2008 article that included the S2PLOT library and that focused on virtual volume rendering in PDF documents. This protocol included for the first time the scripting feature of PDF. The need for commercial software, however, had still not been eliminated. At the beginning of 2012, de Boer and colleagues [60] published a very detailed protocol for the creation of 3D PDFs that described all necessary steps for a basic process and an advanced process on seven pages. It also relied on commercial software (Amira, Adobe Acrobat 9 Pro Extended, Adobe Illustrator), but for the first time it allowed for embedding custom control elements for selecting structures of predefined views of the 3D figure. One month later, Ziegler and colleagues [40] demonstrated how to incorporate a variety of multimedia elements into PDF, including a textured 3D figure (an interactive model of a human face). This publication also mentioned the possibility of using LaTeX to create the final PDF. In August 2011, Danz and Katsaros [61] published their protocol, which was based on a combination of free software (for model generation) and commercial software (for PDF creation). One year later, Shin and colleagues [62] also released a protocol that was still purely based on commercial software; 4 months later, Phelps and colleagues [63] presented another solution that combined a workflow of 22 steps using commercial and noncommercial software. Finally, Lautenschlager [64] published in 2013 another protocol that required a wide range of commercial and noncommercial software.

XSL•FO
RenderX

Table 2. Overview of requirements, restrictions, and possible output of the tools presented in the tool articles. For features see Table 3.

Article	Software needed		Output	Programming needed[a]
	Commercial	Free		
**Articles presenting a protocol**				
Kumar et al [55]	Adobe Acrobat 3D[b], Adobe Acrobat 3D Toolkit[c], Adobe Photoshop, Adobe Illustrator		Model file, final PDF	No
Barnes & Fluke [56]	Adobe Acrobat 3D[b]	S2PLOT	Final PDF	Yes
Ruthensteiner & Heß [57]	Amira, Adobe Acrobat 3D Toolkit[b], Adobe Acrobat 3D[b]		Model file, final PDF	No
Kumar et al [58]	Adobe Acrobat 9 Pro Extended[d], Adobe Acrobat 3D Reviewer[b]	LaTeX, MeshLab	Model file, final PDF	No
de Boer et al [60]	Amira, Adobe Acrobat 9 Pro Extended[d], Adobe Illustrator		Final PDF	No
Ziegler et al [40]	Adobe Acrobat 9 Pro Extended[c,d]	LaTeX	Final PDF	No
Danz & Katsaros [61]	Adobe Acrobat X Pro[d], Tetra 4D 3D PDF Converter plug-in for Acrobat	MeshLab, DAZ Studio		No
Shin et al [62]	Mimics, Autodesk Maya, Deep Exploration[d], Adobe Acrobat 9 Pro Extended[d]		Final PDF	No
Phelps et al [63]	Microsoft PowerPoint, Adobe Acrobat	MeshLab	Model file, final PDF	No
Lautenschlager [64]	Amira, Mimics, Adobe Acrobat 8 Pro[b]/9 Pro Extended[d], Adobe Acrobat 3D Reviewer[b], Adobe Acrobat 3D Toolkit[b], Tetra 4D 3D PDF Converter plug-in for Acrobat, GeoMagic Studio[c], Abaqus FEA[c], Avizo[c], VG Studio Max[c], Autodesk Maya[c]	MeshLab, LaTeX, Drishti[d], SPIERSedit[d], Blender[d], DAZ Studio[d]	Model file, final PDF	No
Mavar-Haramija et al [65]	Amira, Adobe Acrobat 9 Pro Extended[d], Adobe Acrobat 3D Reviewer[b], Tetra 4D 3D PDF Converter plug-in for Acrobat		Model file, final PDF	No
van de Kamp et al [67]	Amira or Avizo, Cinema 4D, Deep Exploration[d], Adobe Acrobat 9 Pro Extended[d]		Model file, final PDF	No
Zhang et al [70]		libHaru	Model file, final PDF	Yes
**Articles presenting a protocol and a library**				
Ruthensteiner et al [59]	Deep Exploration[d], Adobe Acrobat 9 Pro Extended[d]	Amira, S2PLOT, ImageMagick,	Model file, final PDF	Yes
Barnes et al [20]		Asymptote, S2PLOT, LaTeX, libHaru	Model file, final PDF	Yes
**Articles presenting a software app**				
Newe & Ganslandt [66]		MevisLab	Model file	No
Newe [68]		MevisLab	Model file	No
Newe [69]		MevisLab	Model file, final PDF	No
Brandner et al [71]		MevisLab	Model file, final PDF	No

[a]Solution requires the creation of individually tailored software code. Usage of LaTeX is not considered as "programming."

[b]Software is no longer available.

[c]Optional.

[d]Software is no longer available, but a successor which provides the same functionality is available.

XSL•FO
RenderX

Table 3. Overview of features of the tools presented in the tool articles (PDF: Portable Document Format).

Article	Supported geometry type			Supported 3D PDF features					
	Mesh	Polyline	Point cloud	Multiple colors	PMI/markup	Scripting	Texture	Animation	Poster image
**Articles presenting a protocol**									
Kumar et al [55]	x	x	x	x		x	x	x	x
Barnes & Fluke [56]	x	x	x	x		x	x		
Ruthensteiner & Heß [57]	x	x	x	x		x	x	x	x
Kumar et al [58]	x			x		x			
de Boer et al [60]	x			x	x	x	x		
Ziegler et al [40]	x	x	x	x	x	x	x		
Danz & Katsaros [61]	x								
Shin et al [62]	x			x			x		
Phelps et al [63]	x			x					
Lautenschlager [64]	x			x			x	x	x
Mavar-Haramija et al [65]	x	x	x	x		x			
van de Kamp et al [67]	x			x				x	
Zhang et al [70]	x	x	x	x					
**Articles presenting a protocol and a library**									
Ruthensteiner et al [59]	x	x	x	x	x	x	x		x
Barnes et al [20]	x	x	x	x	x	x	x	x	
**Articles presenting a software app**									
Newe & Ganslandt [66]	x			x					
Newe [68]	x	x	x	x					
Newe [69]	x	x	x	x					x
Brandner et al [71]	x	x	x	x			x		x

The first solution that could be used without any commercial software was presented by Barnes and colleagues [20] in September 2013 (ie, 5 years after the first appearance of 3D PDF in biomedical publications). It was based on a number of free software tools and libraries (Asymptote [226]; S2PLOT; LaTeX, libHaru [227]). Although this solution could be used to create both model files and final PDFs, and it could be used free of charge, it had a major drawback: it required writing a program (ie, a certain set of programming skills was necessary to apply this protocol). On the other hand, this gave the user greater flexibility because the program code could be adapted exactly to their needs and to the specific-use case.

Only 2 months later, two more articles with tools for the creation of 3D PDFs were published. Mavar-Haramija and colleagues [4] presented another commercial software-based protocol that focused on control elements for a more sophisticated interaction with the 3D model. At the same time, Newe and Ganslandt [66] presented a noncommercial solution for the creation of model files of surface meshes in U3D format that did not require programming skills. The final PDF could not be created with that solution, but it reduced the number of steps for the creation of such model files and provided a convenient graphical user interface. However, this solution required the installation of the third-party biomedical-imaging framework, MeVisLab [228,229].

In September 2014, a previously unregarded feature of 3D PDF emerged through a new protocol: animation. Van de Kamp and colleagues [67] for the first time demonstrated the feasibility and the usefulness of animated 3D figures in electronic publications. Eight months later, Newe [68] presented an update to his previous publication that provided an advanced user interface for more flexibility. Furthermore, it provided the possibility to embed both point clouds and line sets into the interactive 3D figures. Previous publications usually only considered objects that consisted of surface meshes. However, it was still limited to the creation of model files. The creation of final PDFs was not possible.

The first all-in-one solution for the creation of both model files and the final PDF that did not require any programming was also presented by Newe [69]. The Scientific3DFigurePDFApp provided a graphical user interface for the assembly of 3D models (meshes, line set, point clouds) and included an editor for predefined views. It was also capable of producing one-paged PDF files with the embedded 3D figure that could directly be used as supplementary figures for scientific publications.

XSL•FO
RenderX

The latest complete protocol was published by Zhang and colleagues [70] in November 2017. Although it did not require commercial software, it was also based on the libHaru library (ie, it required the writing of program code). However, this publication featured a deep insight into the U3D file format.

Finally, Brandner and colleagues [71] presented an extension to the toolbox described in [69], which added the support of textures.

Discussion

Summary of Main Findings

With this systematic review, we give an overview of the usage of 3D PDF in the biomedical domain with a special focus on clinical applications. Furthermore, we investigate which protocols and software tools for 3D PDF creation have been published so far. In addition, we hypothesize about possible barriers that might be responsible for the low dissemination of 3D PDF in clinical communication and biomedical sciences. Our primary search revealed 19 tool articles that can be considered as the roots of 3D PDF in biomedical publishing. For these articles, the scope has intentionally not been limited to the biomedical domain, because many biomedical publications seem to be inspired by the Barnes [56] publication (ie, an astronomy publication). Excluding this paper would have left several in-scope biomedical articles unrevealed (eg, [73,88,121,128,131]).

After the secondary search, in which we searched for articles that cited these tool articles, 200 articles were found overall that fulfilled the inclusion criteria and entered into further analysis. This is a very small number with respect to the fact that in the last 10 years (January 1, 2008 to January 1, 2018) a total number of 9,705,959 articles were indexed by PubMed. Even if only 1% of these articles touched the subject of 3D data, 200 articles is still a tiny fraction.

Within these 200 articles, actual 3D PDF figures were available in 156 articles (78.0%). However, the 3D content was embedded directly into the PDF version of the articles in 34 cases only. One reason for this might have been that the journals did not support the direct embedding. This matches the experience of the authors. Even some modern, online-only journals are not capable (and, in some cases, not willing) to provide the possibility to embed 3D figures directly into the PDF versions of their articles.

Possible Reasons for the Low Dissemination of Three-Dimensional Portable Document Format in Medicine and Biomedical Research

As mentioned previously, one reason might be that the journals do not support 3D PDFs. However, a further—and probably more serious reason—might be that most clinicians and scientists do not know about this feature. Although this hypothesis could not be investigated with our systematic review and it should be investigated in future research, the pure existence of this review article might help to draw attention to this technology and help to overcome this threshold.

A further reason might be that the potential target audience—although they know about 3D PDF in principle—do not know how to create 3D PDFs or that they do not have appropriate technical skills. In our systematic review, we reported the protocols and software tools that were used in the 156 articles that apply 3D technology in PDFs. Unfortunately, many of them are based on software packages that are no longer available. Furthermore, the usage of most of the reported protocols or tools needed either programming skills, advanced technical knowledge, and/or expensive, highly specialized, commercial software. Although this gives the user great flexibility because the program code can be adapted exactly to the needs and to the specific-use case, we conclude that the threshold for creating 3D PDFs is still too high and that easy-to-use, standalone software tools are needed to facilitate 3D PDF creation. Of all the software tools analyzed in this review, the solution presented by Newe [69] comes closest to this requirement because it is an all-in-one solution for the creation of both model files and final PDFs, which is operated via a graphical user interface. The major drawback of this solution is the need for the software MeVisLab, which also requires some basic training.

Potential of Three-Dimensional Portable Document Format and Future Research

Although it is not widely disseminated yet, the first approaches are very promising and show that 3D PDF has a high potential in clinical communication, in biomedical research, and in research in general. 3D PDF offers the possibility to simply distribute atlases of, for example, human or animal anatomy as demonstrated in the 3D Atlas of Human Embryology [110,200,230] or in the Visible Korean anatomy database [62,231]. It is, therefore, an alternative to traditional 2D atlases in which much of information gets lost because of the projection of a 3D object to a 2D plane [18]. Furthermore, 3D PDFs can easily be distributed via the internet or shared via emails, making it unnecessary to travel to museums all around the world, for example.

Future research should mainly focus on the development of software tools that can be used easily by everyone and without charge. Furthermore, it should be investigated (eg, by means of surveys) how well-known 3D PDF is in the biomedical and clinical community. Besides, simple instruction manuals for the existing tools are needed and should be disseminated further. Additionally, the routine usage of 3D PDF in clinical applications should be further promoted and evaluated for other disciplines than liver surgery planning. Finally, future research could also consider other domains than the biomedical one.

Limitations

Our review is subject to some limitations. First, an unknown number of articles that would fulfill the inclusion criteria may not have been found. The most probable reason for missing such an article might be that authors developed the 3D PDF on their own (without any of the tool articles) and that the availability of a 3D PDF was not necessarily pointed out in the abstract or in the available full text. Another reason might be that a tool article was used, but not properly cited. However, 200 articles can be considered a solid basis for a review.

XSL•FO
RenderX

Based on our expertise and the high relevance of 3D PDFs in the investigated domain, the scope of this review was intentionally limited to biomedical publications. Other scientific disciplines are known to use 3D PDF as well (eg, astronomy [232], paleontology [233,234], and chemistry [235]) and may yield different results.

Second, as already mentioned, many of the protocols presented previously are based on commercial software that is no longer available and that has no successor providing the same functionality. To cover all available articles, they are still presented, but the use of the more modern protocols is recommended.

Third, a systematic review is not a suitable method to investigate the personal reasons for researchers not using 3D PDF. Other methods such as surveys are needed for this.

Conclusions

The use of 3D PDF for visualization purposes in real medical use cases or in biomedical publications is not yet fully accepted, although the necessary technique is available and there are many arguments in favor of 3D PDF. In this review paper, a wide range of examples for applied 3D PDF technology and a variety of protocols and software tools for the creation of relevant documents and files are presented. It aims to draw attention to this valuable technology, to demonstrate the possibilities, to help interested readers find a suitable solution, and lower the threshold for its use.

Acknowledgments

We acknowledge support by Deutsche Forschungsgemeinschaft (DFG, German Research Foundation) and Friedrich-Alexander University Erlangen-Nürnberg within the funding program Open Access Publishing. Linda Becker was supported by the Bavarian Equal Opportunities Sponsorship—Förderung von Frauen in Forschung und Lehre (FFL)—Promoting Equal Opportunities for Women in Research and Teaching.

Authors' Contributions

AN and LB conceived the study. AN performed the literature search. AN and LB screened the search results. AN and LB analyzed the search results. AN and LB drafted and wrote the manuscript.

Conflicts of Interest

None declared.

Multimedia Appendix 1

3D PDF version of Figure 1.

[PDF File (Adobe PDF File), 3MB - medinform_v6i3e10295_app1.pdf]

Multimedia Appendix 2

3D PDF version of article.

[PDF File (Adobe PDF File), 3MB - medinform_v6i3e10295_app2.pdf]

Multimedia Appendix 3

Overview over the search strategy and all articles that were found and processed.

[XLSX File (Microsoft Excel File), 143KB - medinform_v6i3e10295_app3.xlsx]

Multimedia Appendix 4

Detailed analysis of all eligible articles.

[XLSX File (Microsoft Excel File), 68KB - medinform_v6i3e10295_app4.xlsx]

Multimedia Appendix 5

Detailed analysis of articles that present a protocol or a software for 3D PDF creation.

[XLSX File (Microsoft Excel File), 16KB - medinform_v6i3e10295_app5.xlsx]

References

XSL•FO
RenderX

1. International Organization for Standardization. 2017. ISO 32000-2:2017 Document management--portable document format--Part 2: PDF 2.0 URL: https://www.iso.org/standard/63534.html [accessed 2018-03-01] [WebCite Cache ID 6xb077p9M]
2. Rico RD, Juanes Méndez JA, Mavar-Haramija M, Reina Perticone MA, Prats-Galino A. Possibilities of application of 3D-PDF documents to represent models and tridimensional images in medicine. 2014 Presented at: Second International Conference on Technological Ecosystems for Enhancing Multiculturality; Oct 1-3, 2014; Salamanca, Spain p. 53-56. [doi: 10.1145/2669711.2669878]
3. Newe A, Becker L, Schenk A. Application and evaluation of interactive 3D PDF for presenting and sharing planning results for liver surgery in clinical routine. PLoS One 2014;9(12):e115697 [FREE Full text] [doi: 10.1371/journal.pone.0115697] [Medline: 25551375]
4. Mavar-Haramija M, Prats-Galino A, Méndez JA, Puigdelívoll-Sánchez A, de Notaris M. Interactive 3D-PDF presentations for the simulation and quantification of extended endoscopic endonasal surgical approaches. J Med Syst 2015 Oct;39(10):127. [doi: 10.1007/s10916-015-0282-7] [Medline: 26306875]
5. ASTM International. 2008 Mar 01. PDF Healthcare best practices guide published URL: https://www.astm.org/newsroom/pdf-healthcare-best-practices-guide-published [accessed 2018-03-01] [WebCite Cache ID 6xazlrn0L]
6. Association for Information and Image Management. 2008. Best practices-implementation guide for the portable document format healthcare URL: http://www.aiim.org/documents/standards/PDF-h_Implementation_Guide_2008.pdf [accessed 2018-05-01] [WebCite Cache ID 6QkHzfRns]
7. Adobe Systems Incorporated. 2008 Jul 01. Document management—Portable document format—Part 1: PDF 1.7 URL: http://wwwimages.adobe.com/content/dam/Adobe/en/devnet/pdf/pdfs/PDF32000_2008.pdf [accessed 2018-05-01] [WebCite Cache ID 6S8xkrvPw]
8. International Organization for Standardization. 2013. ISO 32000-1:2008 Document management--portable document format--Part 1: PDF 1.7 URL: http://www.iso.org/iso/iso_catalogue/catalogue_tc/catalogue_detail.htm?csnumber=51502 [accessed 2018-05-01] [WebCite Cache ID 6QkFZgVk9]
9. PDF Association. PDF 2.0: The worldwide standard for electronic documents has evolved URL: https://www.pdfa.org/intro/pdf-2-0-the-worldwide-standard-for-electronic-documents-has-evolved/ [accessed 2018-05-01] [WebCite Cache ID 6xb0LmX8M]
10. Apple. 2004 May 27. MacOS X: System overview: the graphics and windowing environment URL: https://web.archive.org/web/20040925095929/http://developer.apple.com/documentation/MacOSX/Conceptual/SystemOverview/SystemArchitecture/chapter_3_section_4.html [accessed 2018-05-01] [WebCite Cache ID 707QBVPLP]
11. Adobe Systems Incorporated. PDF Reference, Fifth Edition, Adobe Portable Document Format, Version 1.6. 2004. URL: https://wwwimages2.adobe.com/content/dam/acom/en/devnet/pdf/pdf_reference_archive/PDFReference16.pdf [accessed 2018-05-01] [WebCite Cache ID 6xb0tF2Lx]
12. 3dtest. 2004 Mar 11. Q&A: Richard D Benoit, Intel URL: http://www.3d-test.com/contenu.php?id=418 [accessed 2018-05-01] [WebCite Cache ID 6xb18rG94]
13. ECMA International. Standard ECMA-363, Universal 3D File Format, 4th edition. Geneva: Ecma International; 2007 Jun. URL: http://www.ecma-international.org/publications/files/ECMA-ST/ECMA-363%204th%20Edition.pdf [accessed 2018-05-01] [WebCite Cache ID 6QkI44nqn]
14. Dakan M. Acrobat Insider for AEC Professionals. 2006 Apr. Enter the third dimension: Acrobat 7.0 Professional makes working with 3D content easy and convenient URL: http://www.cadalyst.com/aec/acrobat-insider-9-pdf-4614 [accessed 2018-06-12] [WebCite Cache ID 707QUWbUJ]
15. Adobe Systems Incorporated. Adobe Supplement to the ISO 32000, BaseVersion: 1.7, ExtensionLevel: 3. 2008 Jun. URL: http://wwwimages.adobe.com/content/dam/Adobe/en/devnet/pdf/pdfs/adobe_supplement_iso32000.pdf [accessed 2018-05-01] [WebCite Cache ID 6xaz93gNe]
16. Vora K. Adobe Conversations. 2010 May 24. 3D Solutions update URL: https://blogs.adobe.com/conversations/2010/05/3d_solutions_update.html [accessed 2018-03-01] [WebCite Cache ID 6xaz2a98d]
17. Adobe Systems Incorporated. PDF Reference, Second edition, Adobe Portable Document Format, Version 1.3. URL: https://wwwimages2.adobe.com/content/dam/acom/en/devnet/pdf/pdfs/pdf_reference_archives/PDFReference13.pdf [accessed 2018-05-01] [WebCite Cache ID 6xb0wzaki]
18. Tory M, Möller T. Human factors in visualization research. IEEE Trans Vis Comput Graph 2004;10(1):72-84. [doi: 10.1109/TVCG.2004.1260759] [Medline: 15382699]
19. Murienne J, Ziegler A, Ruthensteiner B. A 3D revolution in communicating science. Nature 2008 May 22;453(7194):450. [doi: 10.1038/453450d] [Medline: 18497796]
20. Barnes DG, Vidiassov M, Ruthensteiner B, Fluke CJ, Quayle MR, McHenry CR. Embedding and publishing interactive, 3-dimensional, scientific figures in Portable Document Format (PDF) files. PLoS One 2013;8(9):e69446 [FREE Full text] [doi: 10.1371/journal.pone.0069446] [Medline: 24086243]
21. Maunsell J. Announcement regarding supplemental material. J Neurosci 2010;30(32):10599-10600 [FREE Full text]
22. Elsevier. Interactive U3D models URL: https://www.elsevier.com/authors/author-services/enrichments/interactive-u3d-models [accessed 2018-03-01] [WebCite Cache ID 6xazzYWWN]

XSL•FO
RenderX

23. Aalbersberg IJ, Cos Alvarez P, Jomier J, Marion C, Zudilova-Seinstra E. Bringing 3D visualization into the online research article. ISU 2014 Sep 19;34(1-2):27-37. [doi: 10.3233/ISU-140721]
24. NCBI. PubMed URL: https://www.ncbi.nlm.nih.gov/pubmed [accessed 2018-06-12] [WebCite Cache ID 707iIG5La]
25. Google Scholar. URL: https://scholar.google.de/ [accessed 2018-06-12] [WebCite Cache ID 707iKPOlo]
26. Moher D, Liberati A, Tetzlaff J, Altman DG. Preferred reporting items for systematic reviews and meta-analyses: the PRISMA statement. PLoS Med 2009 Jul 21;6(7):e1000097 [FREE Full text] [doi: 10.1371/journal.pmed.1000097] [Medline: 19621072]
27. Tabernero RR, Juanes MJ, Prats GA. New generation of three-dimensional tools to learn anatomy. J Med Syst 2017 May;41(5):88. [doi: 10.1007/s10916-017-0725-4] [Medline: 28405946]
28. Handschuh S, Schwaha T, Metscher BD. Showing their true colors: a practical approach to volume rendering from serial sections. BMC Dev Biol 2010 Apr 21;10:41 [FREE Full text] [doi: 10.1186/1471-213X-10-41] [Medline: 20409315]
29. Prilusky J, Hodis E, Canner D, Decatur WA, Oberholser K, Martz E, et al. Proteopedia: a status report on the collaborative, 3D web-encyclopedia of proteins and other biomolecules. J Struct Biol 2011 Aug;175(2):244-252. [doi: 10.1016/j.jsb.2011.04.011]
30. Ziegler A. Broad application of non-invasive imaging techniques to echinoids and other echinoderm taxa. Zoosymposia 2012;7(1):53-70. [doi: 10.11646/zoosymposia.7.1.6]
31. Webb HK, Boshkovikj V, Fluke CJ, Truong VK, Hasan J, Baulin VA, et al. Bacterial attachment on sub-nanometrically smooth titanium substrata. Biofouling 2013;29(2):163-170. [doi: 10.1080/08927014.2012.757697] [Medline: 23327438]
32. Margulies DS, Böttger J, Watanabe A, Gorgolewski KJ. Visualizing the human connectome. Neuroimage 2013 Oct 15;80:445-461 [FREE Full text] [doi: 10.1016/j.neuroimage.2013.04.111] [Medline: 23660027]
33. Breure L, Hoogerwerf M, van Horik R. Xpos're: a tool for rich Internet publications. Digit Hum Quart 2014;8(2).
34. Malkowsky Y, Jochum A. Three-dimensional reconstructions of pallial eyes in Pectinidae (Mollusca: Bivalvia). Acta Zool 2014 Jan 31;96(2):167-173. [doi: 10.1111/azo.12064]
35. Carbayo F, Francoy TM, Giribet G. Non-destructive imaging to describe a new species of land planarian (Platyhelminthes, Tricladida). Zool Scr 2016 Mar 03;45(5):566-578. [doi: 10.1111/zsc.12175]
36. Chung BS, Kim J, Chung MS. Integrated comics and visible Korean movies for laypeople's learning of systemic anatomy. Int J Morphol 2017 Sep;35(3):883-887. [doi: 10.4067/S0717-95022017000300014]
37. Laaß M, Schillinger B, Werneburg I. Neutron tomography and X-ray tomography as tools for the morphological investigation of non-mammalian Synapsids. Physics Procedia 2017;88:100-108. [doi: 10.1016/j.phpro.2017.06.013]
38. Schachner ER, Sedlmayr JC, Schott R, Lyson TR, Sanders RK, Lambertz M. Pulmonary anatomy and a case of unilateral aplasia in a common snapping turtle (Chelydra serpentina): developmental perspectives on cryptodiran lungs. J Anat 2017 Dec;231(6):835-848. [doi: 10.1111/joa.12722] [Medline: 29063595]
39. Chung BS, Chung MS. Homepage to distribute the anatomy learning contents including Visible Korean products, comics, and books. Anat Cell Biol 2018 Mar;51(1):7-13 [FREE Full text] [doi: 10.5115/acb.2018.51.1.7] [Medline: 29644104]
40. Ziegler A, Mietchen D, Faber C, von Hausen W, Schöbel C, Sellerer M, et al. Effectively incorporating selected multimedia content into medical publications. BMC Med 2011 Feb 17;9:17 [FREE Full text] [doi: 10.1186/1741-7015-9-17] [Medline: 21329532]
41. Mavar-Haramija M, Prats-Galino A, Méndez J, Puigdelívoll-Sánchez A, de Notaris M. Simulation of surgical procedures and associated quantification methods by 3D PDF documents. 2014 Presented at: Second International Conference on Technological Ecosystems for Enhancing Multiculturality; Oct 1-3, 2014; Salamanca, Spain p. 57-62. [doi: 10.1145/2669711.2669879]
42. Park JS, Chung BS, Chung MS. Digital anatomy using the surface models in portable document format file for self-learning and evaluation. Digit Med 2017;3(3):133. [doi: 10.4103/digm.digm_29_17]
43. Wirkner CS, Richter S. Evolutionary morphology of the circulatory system in Peracarida (Malacostraca; Crustacea). Cladistics 2009;26(2):143-167. [doi: 10.1111/j.1096-0031.2009.00278.x]
44. Ziegler A, Kunth M, Mueller S, Bock C, Pohmann R, Schröder L, et al. Application of magnetic resonance imaging in zoology. Zoomorphology 2011 Oct 13;130(4):227-254. [doi: 10.1007/s00435-011-0138-8]
45. Ziegler A, Stock SR, Menze BH, Smith AB. Macro- and microstructural diversity of sea urchin teeth revealed by large-scale mircro-computed tomography survey. In: Proc SPIE 8506: Developments in X-Ray Tomography VIII. 2012 Presented at: SPIE Optical Engineering + Applications; Oct 17, 2012; San Diego, CA. [doi: 10.1117/12.930832]
46. Gaweł D, Danielewicz K, Nowak M. The method of geometrical comparison of 3-dimensional objects created from DICOM images. Stud Health Technol Inform 2012;176:273-276. [Medline: 22744507]
47. van de Kamp T, Ershov A, Dos Santos Rolo T, Riedel A, Baumbach T. Insect imaging at the ANKA synchrotron radiation facility. Entomologie heute 2013;25:147-160.
48. Lautenschlager S, Rücklin M. Beyond the print-virtual paleontology in science publishing, outreach, and education. J Paleontol 2014 Jul 01;88(4):727-734 [FREE Full text] [doi: 10.1666/13-085] [Medline: 26306051]
49. Jocks I, Livingstone D, Rea PM. An investigation to examine the most appropriate methodology to capture historical and modern preserved anatomical specimens for use in the digital age to improve access: a pilot study. In: Proceedings of

XSL•FO
RenderX

INTED2015 Conference. 2015 Presented at: 9th International Technology, Education and Development Conference; Mar 2-4, 2015; Madrid, Spain p. 6377-6386.
50. Xavier JC, Allcock AL, Cherel Y, Lipinski MR, Pierce GJ, Rodhouse PG, et al. Future challenges in cephalopod research. J Mar Biol Assoc 2014 Jun 26;95(05):999-1015. [doi: 10.1017/S0025315414000782]
51. Newe A, Becker L. Using interactive 3D PDF for exploring complex biomedical data: experiences and solutions. Stud Health Technol Inform 2016;228:740-744. [doi: 10.3233/978-1-61499-678-1-740] [Medline: 27577484]
52. Williston AD. Charles Minot and the Harvard Embryological Collection: over a century of development. Breviora 2016 Apr;547(1):1-13. [doi: 10.3099/0006-9698-547.00.1]
53. Lebrun R, Orliac MJ. Morphomuseum: an online platform for publication and storage of virtual specimens. Paleontol Soc Pap 2017 Apr 27;22:183-195. [doi: 10.1017/scs.2017.14]
54. Elbashti ME, Aswehlee AM, Sumita YI, Hattori M, Taniguchi H. The role of portable documentation format in three-dimensional interactive visualization in maxillofacial prosthetics. Int J Prosthodont 2018 Mar 28. [doi: 10.11607/ijp.5475] [Medline: 29590664]
55. Kumar P, Ziegler A, Ziegler J, Uchanska-Ziegler B, Ziegler A. Grasping molecular structures through publication-integrated 3D models. Trends Biochem Sci 2008 Sep;33(9):408-412. [doi: 10.1016/j.tibs.2008.06.004] [Medline: 18672371]
56. Barnes DG, Fluke CJ. Incorporating interactive three-dimensional graphics in astronomy research papers. New Astron 2008 Nov;13(8):599-605. [doi: 10.1016/j.newast.2008.03.008]
57. Ruthensteiner B, Hess M. Embedding 3D models of biological specimens in PDF publications. Microsc Res Tech 2008 Nov;71(11):778-786. [doi: 10.1002/jemt.20618] [Medline: 18785246]
58. Kumar P, Ziegler A, Grahn A, Hee CS, Ziegler A. Leaving the structural ivory tower, assisted by interactive 3D PDF. Trends Biochem Sci 2010 Aug;35(8):419-422. [doi: 10.1016/j.tibs.2010.03.008] [Medline: 20541422]
59. Ruthensteiner B, Baeumler N, Barnes DG. Interactive 3D volume rendering in biomedical publications. Micron 2010 Oct;41(7):886.e1-886.e17. [doi: 10.1016/j.micron.2010.03.010] [Medline: 20562000]
60. de Boer BA, Soufan AT, Hagoort J, Mohun TJ, van den Hoff MJ, Hasman A, et al. The interactive presentation of 3D information obtained from reconstructed datasets and 3D placement of single histological sections with the 3D portable document format. Development 2011 Jan;138(1):159-167 [FREE Full text] [doi: 10.1242/dev.051086] [Medline: 21138978]
61. Danz JC, Katsaros C. Three-dimensional portable document format: a simple way to present 3-dimensional data in an electronic publication. Am J Orthod Dentofacial Orthop 2011 Aug;140(2):274-276. [doi: 10.1016/j.ajodo.2011.04.010] [Medline: 21803267]
62. Shin DS, Chung MS, Park JS, Park HS, Lee S, Moon YL, et al. Portable document format file showing the surface models of cadaver whole body. J Korean Med Sci 2012 Aug;27(8):849-856 [FREE Full text] [doi: 10.3346/jkms.2012.27.8.849] [Medline: 22876049]
63. Phelps A, Naeger DM, Marcovici P. Embedding 3D radiology models in portable document format. AJR Am J Roentgenol 2012 Dec;199(6):1342-1344. [doi: 10.2214/AJR.12.8716] [Medline: 23169728]
64. Lautenschlager S. Palaeontology in the third dimension: a comprehensive guide for the integration of three-dimensional content in publications. Paläontol Z 2013 May 31;88(1):111-121. [doi: 10.1007/s12542-013-0184-2]
65. Mavar-Haramija M, Prats-Galino A, Escuder CB, Juanes Méndez JA, Puigdelívoll-Sánchez A. 3D PDF Technology combined with JavaScript functions enables the creation and visualization of interactive 3D presentations. 2013 Presented at: First International Conference on Technological Ecosystem for Enhancing Multiculturality; Nov 14-15, 2013; Salamanca, Spain p. 67-72. [doi: 10.1145/2536536.2536548]
66. Newe A, Ganslandt T. Simplified generation of biomedical 3D surface model data for embedding into 3D portable document format (PDF) files for publication and education. PLoS One 2013;8(11):e79004 [FREE Full text] [doi: 10.1371/journal.pone.0079004] [Medline: 24260144]
67. van de Kamp T, dos Santos Rolo T, Vagovič P, Baumbach T, Riedel A. Three-dimensional reconstructions come to life--interactive 3D PDF animations in functional morphology. PLoS One 2014;9(7):e102355 [FREE Full text] [doi: 10.1371/journal.pone.0102355] [Medline: 25029366]
68. Newe A. Towards an easier creation of three-dimensional data for embedding into scholarly 3D PDF (Portable Document Format) files. PeerJ 2015;3:e794 [FREE Full text] [doi: 10.7717/peerj.794] [Medline: 25780759]
69. Newe A. Enriching scientific publications with interactive 3D PDF: an integrated toolbox for creating ready-to-publish figures. PeerJ Comp Sci 2016;2:e64. [doi: 10.7717/peerj-cs.64]
70. Zhang N, Li Q, Jia H, Zhang M. U3D File format analyzing and 3DPDF generating method. In: Wang Y, editor. Advances in Image and Graphics Technologies. IGTA 2017. Communications in Computer and Information Science, vol 757. Singapore: Springer; 2017:136-146.
71. Brandner J, Newe A, Aichinger W, Linda B. An open source tool for creating model files for virtual volume rendering in PDF documents. In: Maier A, Deserno T, Handels H, Maier-Hein K, Palm C, Tolxdorff T, editors. Bildverarbeitung für die Medizin 2018. Informatik aktuell. Berlin: Springer; 2018:133-138.
72. Ganesan A, Wang F, Falzon C. Intramolecular interactions of L-phenylalanine: valence ionization spectra and orbital momentum distributions of its fragment molecules. J Comput Chem 2011 Feb;32(3):525-535. [doi: 10.1002/jcc.21639] [Medline: 20806261]

XSL•FO
RenderX

73. Truong VK, Lapovok R, Estrin YS, Rundell S, Wang JY, Fluke CJ, et al. The influence of nano-scale surface roughness on bacterial adhesion to ultrafine-grained titanium. Biomaterials 2010 May;31(13):3674-3683. [doi: 10.1016/j.biomaterials.2010.01.071] [Medline: 20163851]
74. Strickfaden H, Zunhammer A, van Koningsbruggen S, Köhler D, Cremer T. 4D chromatin dynamics in cycling cells: Theodor Boveri's hypotheses revisited. Nucleus 2010;1(3):284-297 [FREE Full text] [doi: 10.4161/nucl.1.3.11969] [Medline: 21327076]
75. Loll B, Rückert C, Hee CS, Saenger W, Uchanska-Ziegler B, Ziegler A. Loss of recognition by cross-reactive T cells and its relation to a C-terminus-induced conformational reorientation of an HLA-B*2705-bound peptide. Protein Sci 2011 Feb;20(2):278-290 [FREE Full text] [doi: 10.1002/pro.559] [Medline: 21280120]
76. Lintner NG, Frankel KA, Tsutakawa SE, Alsbury DL, Copié V, Young MJ, et al. The structure of the CRISPR-associated protein Csa3 provides insight into the regulation of the CRISPR/Cas system. J Mol Biol 2011 Jan 28;405(4):939-955 [FREE Full text] [doi: 10.1016/j.jmb.2010.11.019] [Medline: 21093452]
77. Uchanska-Ziegler B, Loll B, Fabian H, Hee CS, Saenger W, Ziegler A. HLA class I-associated diseases with a suspected autoimmune etiology: HLA-B27 subtypes as a model system. Eur J Cell Biol 2012 Apr;91(4):274-286. [doi: 10.1016/j.ejcb.2011.03.003] [Medline: 21665321]
78. Hee CS, Fabian H, Uchanska-Ziegler B, Ziegler A, Loll B. Comparative biophysical characterization of chicken β2-microglobulin. Biophys Chem 2012 Jun;167:26-35. [doi: 10.1016/j.bpc.2012.04.001] [Medline: 22695053]
79. Balke M, Ruthensteiner B, Warikar EL, Neven K, Hendrich L. Two new species of Limbodessus diving beetles from New Guinea - short verbal descriptions flanked by online content (digital photography, μCT scans, drawings and DNA sequence data). Biodivers Data J 2015(3):e7096 [FREE Full text] [doi: 10.3897/BDJ.3.e7096] [Medline: 26752969]
80. Chung BS, Kwon K, Shin BS, Chung MS. Peeled and piled volume models of the stomach made from a cadaver's sectioned images. Int J Morphol 2016 Sep;34(3):939-944. [doi: 10.4067/S0717-95022016000300020]
81. Shin DS, Park SK. Surface reconstruction and optimization of cerebral cortex for application use. J Craniofac Surg 2016 Mar;27(2):489-492. [doi: 10.1097/SCS.0000000000002352] [Medline: 26854785]
82. Baeumler N, Haszprunar G, Ruthensteiner B. 3D interactive microanatomy of Omalogyra atomus (Philippi, 1841) (Gastropoda, Heterobranchia, Omalogyridae). Zoosymposia 2008;1:101-118. [doi: 10.11646/zoosymposia.1.1.9]
83. Ziegler A, Faber C, Mueller S, Bartolomaeus T. Systematic comparison and reconstruction of sea urchin (Echinoidea) internal anatomy: a novel approach using magnetic resonance imaging. BMC Biol 2008 Jul 23;6:33 [FREE Full text] [doi: 10.1186/1741-7007-6-33] [Medline: 18651948]
84. Kumar P, Vahedi-Faridi A, Saenger W, Ziegler A, Uchanska-Ziegler B. Conformational changes within the HLA-A1:MAGE-A1 complex induced by binding of a recombinant antibody fragment with TCR-like specificity. Protein Sci 2009 Jan;18(1):37-49 [FREE Full text] [doi: 10.1002/pro.4] [Medline: 19177349]
85. Selvam L, Vasilyev V, Wang F. Methylation of zebularine: a quantum mechanical study incorporating interactive 3D pdf graphs. J Phys Chem B 2009 Aug 20;113(33):11496-11504. [doi: 10.1021/jp901678g] [Medline: 19637931]
86. Neusser TP, Heß M, Schrödl M. Tiny but complex - interactive 3D visualization of the interstitial acochlidian gastropod Pseudunela cornuta (Challis, 1970). Front Zool 2009 Sep 11;6:20 [FREE Full text] [doi: 10.1186/1742-9994-6-20] [Medline: 19747373]
87. Kumar P, Vahedi-Faridi A, Saenger W, Merino E, López de Castro JA, Uchanska-Ziegler B, et al. Structural basis for T cell alloreactivity among three HLA-B14 and HLA-B27 antigens. J Biol Chem 2009 Oct 23;284(43):29784-29797 [FREE Full text] [doi: 10.1074/jbc.M109.038497] [Medline: 19617632]
88. Ivanova EP, Truong VK, Wang JY, Berndt CC, Jones RT, Yusuf II, et al. Impact of nanoscale roughness of titanium thin film surfaces on bacterial retention. Langmuir 2010 Feb 02;26(3):1973-1982. [doi: 10.1021/la902623c] [Medline: 19842625]
89. Mitik-Dineva N, Wang J, Truong VK, Stoddart PR, Alexander MR, Albutt DJ, et al. Bacterial attachment on optical fibre surfaces. Biofouling 2010 May;26(4):461-471. [doi: 10.1080/08927011003753399] [Medline: 20358429]
90. Ziegler A, Ogurreck M, Steinke T, Beckmann F, Prohaska S, Ziegler A. Opportunities and challenges for digital morphology. Biol Direct 2010 Jul 06;5:45 [FREE Full text] [doi: 10.1186/1745-6150-5-45] [Medline: 20604956]
91. Ziegler A, Mooi R, Rolet G, De Ridder C. Origin and evolutionary plasticity of the gastric caecum in sea urchins (Echinodermata: Echinoidea). BMC Evol Biol 2010 Oct 18;10:313 [FREE Full text] [doi: 10.1186/1471-2148-10-313] [Medline: 20955602]
92. Ruthensteiner B, Schropel V, Haszprunar G. Anatomy and affinities of Micropilina minuta Waren, 1989 (Monoplacophora: Micropilinidae). J Mollus Stud 2010 Jun 09;76(4):323-332. [doi: 10.1093/mollus/eyq013]
93. Hartmann H, Hess M, Haszprunar G. Interactive 3D anatomy and affinities of Bathysciadiidae (Gastropoda, Cocculinoidea): Deep-sea limpets feeding on decaying cephalopod beaks. J Morphol 2011 Mar;272(3):259-279. [doi: 10.1002/jmor.10910] [Medline: 21312226]
94. Brenzinger B, Neusser TP, Jörger KM, Schrödl M. Integrating 3D microanatomy and molecules: natural history of the Pacific freshwater slug Strubellia Odhner, 1937 (Heterobranchia: Acochlidia), with description of a new species. J Mollus Stud 2011;77(4):351-374. [doi: 10.1093/mollus/eyr027]
95. Klußmann-Fricke B, Prendini L, Wirkner CS. Evolutionary morphology of the hemolymph vascular system in scorpions: a character analysis. Arthropod Struct Dev 2012 Nov;41(6):545-560. [doi: 10.1016/j.asd.2012.06.002] [Medline: 22735399]

XSL•FO
RenderX

96. Lipke E, Michalik P. Formation of primary sperm conjugates in a haplogyne spider (Caponiidae, Araneae) with remarks on the evolution of sperm conjugation in spiders. Arthropod Struct Dev 2012 Nov;41(6):561-573. [doi: 10.1016/j.asd.2012.08.001] [Medline: 22982877]
97. Genolini C, Pingault JB, Driss T, Côté S, Tremblay RE, Vitaro F, et al. KmL3D: a non-parametric algorithm for clustering joint trajectories. Comput Methods Programs Biomed 2013 Jan;109(1):104-111. [doi: 10.1016/j.cmpb.2012.08.016] [Medline: 23127283]
98. Faulwetter S, Vasileiadou A, Kouratoras M, Thanos D, Arvanitidis C. Micro-computed tomography: Introducing new dimensions to taxonomy. Zookeys 2013;4(263):1-45 [FREE Full text] [doi: 10.3897/zookeys.263.4261] [Medline: 23653515]
99. Düring DN, Ziegler A, Thompson CK, Ziegler A, Faber C, Müller J, et al. The songbird syrinx morphome: a three-dimensional, high-resolution, interactive morphological map of the zebra finch vocal organ. BMC Biol 2013 Jan 08;11:1 [FREE Full text] [doi: 10.1186/1741-7007-11-1] [Medline: 23294804]
100. Hawe A, Heß M, Haszprunar G. 3D reconstruction of the anatomy of the ovoviviparous (?) freshwater gastropod Borysthenia naticina (Menke, 1845)(Ectobranchia: Valvatidae). J Mollus Stud 2013;79(3):191-201. [doi: 10.1093/mollus/eyt018]
101. Kato A, Ziegler A, Higuchi N, Nakata K, Nakamura H, Ohno N. Aetiology, incidence and morphology of the C-shaped root canal system and its impact on clinical endodontics. Int Endod J 2014 Nov;47(11):1012-1033 [FREE Full text] [doi: 10.1111/iej.12256] [Medline: 24483229]
102. Haszprunar G, Graf L, Hess M. 3D-anatomy of the ctenoglossate limpet Kaiparapelta (Vetigastropoda: Lepetelloidea). J Mollus Stud 2014 Jan 20;80(1):84-98. [doi: 10.1093/mollus/eyt051]
103. Hawe A, Paroll C, Haszprunar G. Interactive 3D-anatomical reconstruction and affinities of the hot-vent gastropod Xylodiscula analoga Waren & Bouchet, 2001 (Ectobranchia). J Mollus Stud 2014 Apr 08;80(3):315-325. [doi: 10.1093/mollus/eyu017]
104. Judge J, Haszprunar G. The anatomy of Lepetella sierrai (Vetigastropoda, Lepetelloidea): implications for reproduction, feeding, and symbiosis in lepetellid limpets. Invertebr Biol 2014 Jul 02;133(4):324-339. [doi: 10.1111/ivb.12064]
105. Ziegler A. Rediscovery of an internal organ in heart urchins (Echinoidea: Spatangoida): morphology and evolution of the intestinal caecum. Org Divers Evol 2014 Jul 9;14(4):383-395. [doi: 10.1007/s13127-014-0178-2]
106. Ziegler A, Lenihan J, Zachos LG, Faber C, Mooi R. Comparative morphology and phylogenetic significance of Gregory's diverticulum in sand dollars (Echinoidea: Clypeasteroida). Org Divers Evol 2015 Aug 28;16(1):141-166. [doi: 10.1007/s13127-015-0231-9]
107. Kato A, Ziegler A, Utsumi M, Ohno K, Takeichi T. Three-dimensional imaging of internal tooth structures: applications in dental education. J Oral Biosci 2016 Aug;58(3):100-111. [doi: 10.1016/j.job.2016.05.004]
108. Kunze T, Heß M, Haszprunar G. 3D-interactive microanatomy of Ventsia tricarinata Warén & Bouchet, 1993 (Vetigastropoda: Seguenzioidea) from Pacific hydrothermal vents. J Mollus Stud 2016 Feb 17;82(3):366-377. [doi: 10.1093/mollus/eyw002]
109. Day NJ, Earnshaw D, Salazar-Ferrer P, Walsh CJ. Preoperative mapping of fistula-in-ano: a new three-dimensional MRI-based modelling technique. Colorectal Dis 2013 Nov;15(11):e699-e701. [doi: 10.1111/codi.12438] [Medline: 24119050]
110. de Bakker BS, de Jong KH, Hagoort J, Oostra RJ, Moorman AF. Towards a 3-dimensional atlas of the developing human embryo: the Amsterdam experience. Reprod Toxicol 2012 Sep;34(2):225-236. [doi: 10.1016/j.reprotox.2012.05.087] [Medline: 22640940]
111. de Doer BA, van den Berg G, de Boer PAJ, Moorman AFM, Ruijter JM. Growth of the developing mouse heart: an interactive qualitative and quantitative 3D atlas. Dev Biol 2012 Aug 15;368(2):203-213 [FREE Full text] [doi: 10.1016/j.ydbio.2012.05.001] [Medline: 22617458]
112. Holliday CM, Tsai HP, Skiljan RJ, George ID, Pathan S. A 3D interactive model and atlas of the jaw musculature of Alligator mississippiensis. PLoS One 2013;8(6):e62806 [FREE Full text] [doi: 10.1371/journal.pone.0062806] [Medline: 23762228]
113. Lautenschlager S, Bright JA, Rayfield EJ. Digital dissection - using contrast-enhanced computed tomography scanning to elucidate hard- and soft-tissue anatomy in the Common Buzzard Buteo buteo. J Anat 2014 Apr;224(4):412-431 [FREE Full text] [doi: 10.1111/joa.12153] [Medline: 24350638]
114. Quayle MR, Barnes DG, Kaluza OL, McHenry CR. An interactive three dimensional approach to anatomical description-the jaw musculature of the Australian laughing kookaburra (Dacelo novaeguineae). PeerJ 2014;2:e355 [FREE Full text] [doi: 10.7717/peerj.355] [Medline: 24860694]
115. Jensen B, Moorman AF, Wang T. Structure and function of the hearts of lizards and snakes. Biol Rev Camb Philos Soc 2014 May;89(2):302-336. [doi: 10.1111/brv.12056] [Medline: 23998743]
116. Scherz MD, Ruthensteiner B, Vences M, Glaw F. A new microhylid frog, genus Rhombophryne, from northeastern Madagascar, and a re-description of R. serratopalpebrosa using micro-computed tomography. Zootaxa 2014 Sep 10;3860(6):547-560. [doi: 10.11646/zootaxa.3860.6.3] [Medline: 25283290]
117. Xu XG. An exponential growth of computational phantom research in radiation protection, imaging, and radiotherapy: a review of the fifty-year history. Phys Med Biol 2014 Sep 21;59(18):R233-R302 [FREE Full text] [doi: 10.1088/0031-9155/59/18/R233] [Medline: 25144730]

XSL•FO
RenderX

118. Wu Y, Dabhoiwala NF, Hagoort J, Shan JL, Tan LW, Fang BJ, et al. 3D Topography of the young adult anal sphincter complex reconstructed from undeformed serial anatomical sections. PLoS One 2015;10(8):e0132226 [FREE Full text] [doi: 10.1371/journal.pone.0132226] [Medline: 26305117]
119. Prötzel D, Ruthensteiner B, Scherz MD, Glaw F. Systematic revision of the Malagasy chameleons Calumma boettgeri and C. linotum (Squamata: Chamaeleonidae). Zootaxa 2015 Nov 25;4048(2):211-231. [Medline: 26624746]
120. Porro LB, Richards CT. Digital dissection of the model organism Xenopus laevis using contrast-enhanced computed tomography. J Anat 2017 Aug;231(2):169-191. [doi: 10.1111/joa.12625] [Medline: 28547827]
121. Truong VK, Rundell S, Lapovok R, Estrin Y, Wang JY, Berndt CC, et al. Effect of ultrafine-grained titanium surfaces on adhesion of bacteria. Appl Microbiol Biotechnol 2009 Jul;83(5):925-937. [doi: 10.1007/s00253-009-1944-5] [Medline: 19296098]
122. Bangs F, Welten M, Davey MG, Fisher M, Yin Y, Downie H, et al. Identification of genes downstream of the Shh signalling in the developing chick wing and syn-expressed with Hoxd13 using microarray and 3D computational analysis. Mech Dev 2010;127(9-12):428-441 [FREE Full text] [doi: 10.1016/j.mod.2010.08.001] [Medline: 20708683]
123. Hee CS, Gao S, Loll B, Miller MM, Uchanska-Ziegler B, Daumke O, et al. Structure of a classical MHC class I molecule that binds “non-classical” ligands. PLoS Biol 2010 Dec 07;8(12):e1000557 [FREE Full text] [doi: 10.1371/journal.pbio.1000557] [Medline: 21151886]
124. Neusser TP, Jörger KM, Schrödl M. Cryptic species in tropic sands--interactive 3D anatomy, molecular phylogeny and evolution of meiofaunal Pseudunelidae (Gastropoda, Acochlidia). PLoS One 2011;6(8):e23313 [FREE Full text] [doi: 10.1371/journal.pone.0023313] [Medline: 21912592]
125. van den Berg G, Moorman AF. Development of the pulmonary vein and the systemic venous sinus: an interactive 3D overview. PLoS One 2011;6(7):e22055 [FREE Full text] [doi: 10.1371/journal.pone.0022055] [Medline: 21779373]
126. Sizarov A, Ya J, de Boer BA, Lamers WH, Christoffels VM, Moorman AFM. Formation of the building plan of the human heart: morphogenesis, growth, and differentiation. Circulation 2011 Mar 15;123(10):1125-1135 [FREE Full text] [doi: 10.1161/CIRCULATIONAHA.110.980607] [Medline: 21403123]
127. Haszprunar G, Speimann E, Hawe A, Heß M. Interactive 3D anatomy and affinities of the Hyalogyrinidae, basal Heterobranchia (Gastropoda) with a rhipidoglossate radula. Org Divers Evol 2011 Jun 19;11(3):201-236. [doi: 10.1007/s13127-011-0048-0]
128. Ivanova EP, Hasan J, Truong VK, Wang JY, Raveggi M, Fluke C, et al. The influence of nanoscopically thin silver films on bacterial viability and attachment. Appl Microbiol Biotechnol 2011 Aug;91(4):1149-1157. [doi: 10.1007/s00253-011-3195-5] [Medline: 21556922]
129. Sylva M, Li VS, Buffing AA, van Es JH, van den Born M, van der Velden S, et al. The BMP antagonist follistatin-like 1 is required for skeletal and lung organogenesis. PLoS One 2011;6(8):e22616 [FREE Full text] [doi: 10.1371/journal.pone.0022616] [Medline: 21826198]
130. Sizarov A, Devalla HD, Anderson RH, Passier R, Christoffels VM, Moorman AF. Molecular analysis of patterning of conduction tissues in the developing human heart. Circ Arrhythm Electrophysiol 2011 Aug;4(4):532-542 [FREE Full text] [doi: 10.1161/CIRCEP.111.963421] [Medline: 21576278]
131. Ivanova EP, Truong VK, Webb HK, Baulin VA, Wang JY, Mohammodi N, et al. Differential attraction and repulsion of Staphylococcus aureus and Pseudomonas aeruginosa on molecularly smooth titanium films. Sci Rep 2011;1:165 [FREE Full text] [doi: 10.1038/srep00165] [Medline: 22355680]
132. Sizarov A, Lamers WH, Mohun TJ, Brown NA, Anderson RH, Moorman AF. Three-dimensional and molecular analysis of the arterial pole of the developing human heart. J Anat 2012 Apr;220(4):336-349 [FREE Full text] [doi: 10.1111/j.1469-7580.2012.01474.x] [Medline: 22296102]
133. Brand J, Smith ES, Schwefel D, Lapatsina L, Poole K, Omerbašić D, et al. A stomatin dimer modulates the activity of acid-sensing ion channels. EMBO J 2012 Aug 29;31(17):3635-3646 [FREE Full text] [doi: 10.1038/emboj.2012.203] [Medline: 22850675]
134. Baeumler N, Haszprunar G, Ruthensteiner B. Development of the excretory system in a polyplacophoran mollusc: stages in metanephridial system development. Front Zool 2012 Sep 14;9(1):23 [FREE Full text] [doi: 10.1186/1742-9994-9-23] [Medline: 22973977]
135. Deans AR, Mikó I, Wipfler B, Friedrich F. Evolutionary phenomics and the emerging enlightenment of arthropod systematics. Invert Systematics 2012;26(3):323. [doi: 10.1071/IS12063]
136. de Boer BA, van den Berg G, Soufan AT, de Boer PA, Hagoort J, van den Hoff MJ, et al. Measurement and 3D-visualization of cell-cycle length using double labelling with two thymidine analogues applied in early heart development. PLoS One 2012;7(10):e47719 [FREE Full text] [doi: 10.1371/journal.pone.0047719] [Medline: 23091641]
137. Nakamura MJ, Terai J, Okubo R, Hotta K, Oka K. Three-dimensional anatomy of the Ciona intestinalis tailbud embryo at single-cell resolution. Dev Biol 2012 Dec 15;372(2):274-284 [FREE Full text] [doi: 10.1016/j.ydbio.2012.09.007] [Medline: 23022659]
138. Brenzinger B, Padula V, Schrödl M. Insemination by a kiss? Interactive 3D-microanatomy, biology and systematics of the mesopsammic cephalaspidean sea slug Pluscula cuica Marcus, 1953 from Brazil (Gastropoda: Euopisthobranchia: Philinoglossidae). Org Divers Evol 2012 Jun 12;13(1):33-54. [doi: 10.1007/s13127-012-0093-3]

XSL•FO
RenderX

139. Jensen B, van den Berg G, van den Doel R, Oostra RJ, Wang T, Moorman AF. Development of the hearts of lizards and snakes and perspectives to cardiac evolution. PLoS One 2013;8(6):e63651 [FREE Full text] [doi: 10.1371/journal.pone.0063651] [Medline: 23755108]
140. Brenzinger B, Haszprunar G, Schrödl M. At the limits of a successful body plan - 3D microanatomy, histology and evolution of Helminthope (Mollusca: Heterobranchia: Rhodopemorpha), the most worm-like gastropod. Front Zool 2013 Jun 28;10(1):37 [FREE Full text] [doi: 10.1186/1742-9994-10-37] [Medline: 23809165]
141. Giraud-Billoud M, Gamarra-Luques C, Castro-Vazquez A. Functional anatomy of male copulatory organs of Pomacea canaliculata (Caenogastropoda, Ampullariidae). Zoomorphology 2012 Dec 15;132(2):129-143. [doi: 10.1007/s00435-012-0183-y]
142. White MA, Benson RB, Tischler TR, Hocknull SA, Cook AG, Barnes DG, et al. New Australovenator hind limb elements pertaining to the holotype reveal the most complete Neovenatorid leg. PLoS One 2013;8(7):e68649 [FREE Full text] [doi: 10.1371/journal.pone.0068649] [Medline: 23894328]
143. Schulz-Mirbach T, Heß M, Metscher BD, Ladich F. A unique swim bladder-inner ear connection in a teleost fish revealed by a combined high-resolution microtomographic and three-dimensional histological study. BMC Biol 2013 Jul 04;11:75 [FREE Full text] [doi: 10.1186/1741-7007-11-75] [Medline: 23826967]
144. Schulz-Mirbach T, Heß M, Metscher BD. Sensory epithelia of the fish inner ear in 3D: studied with high-resolution contrast enhanced microCT. Front Zool 2013 Oct 27;10(1):63 [FREE Full text] [doi: 10.1186/1742-9994-10-63] [Medline: 24160754]
145. Kohnert P, Brenzinger B, Jensen KR, Schrödl M. 3D- microanatomy of the semiterrestrial slug Gascoignella aprica Jensen, 1985—a basal plakobranchacean sacoglossan (Gastropoda, Panpulmonata). Org Divers Evol 2013 Jun 19;13(4):583-603. [doi: 10.1007/s13127-013-0142-6]
146. Schulz-Mirbach T, Ladich F, Plath M, Metscher BD, Heß M. Are accessory hearing structures linked to inner ear morphology? Insights from 3D orientation patterns of ciliary bundles in three cichlid species. Front Zool 2014 Mar 19;11(1):25 [FREE Full text] [doi: 10.1186/1742-9994-11-25] [Medline: 24645675]
147. Hawe A, Haszprunar G. 3D-microanatomy and histology of the hydrothermal vent gastropod Lurifax vitreus Warén & Bouchet, 2001 (Heterobranchia: Orbitestellidae) and comparisons with Ectobranchia. Org Divers Evol 2014;14:43-55. [doi: 10.1007/s13127-013-0155-1]
148. Fernández R, Kvist S, Lenihan J, Giribet G, Ziegler A. Sine systemate chaos? A versatile tool for earthworm taxonomy: non-destructive imaging of freshly fixed and museum specimens using micro-computed tomography. PLoS One 2014;9(5):e96617 [FREE Full text] [doi: 10.1371/journal.pone.0096617] [Medline: 24837238]
149. Cox PG, Faulkes CG. Digital dissection of the masticatory muscles of the naked mole-rat, Heterocephalus glaber (Mammalia, Rodentia). PeerJ 2014;2:e448 [FREE Full text] [doi: 10.7717/peerj.448] [Medline: 25024917]
150. Sizarov A, de Bakker BS, Klein K, Ohlerth S. Building foundations for transcatheter intervascular anastomoses: 3D anatomy of the great vessels in large experimental animals. Interact Cardiovasc Thorac Surg 2014 Oct;19(4):543-551. [doi: 10.1093/icvts/ivu210] [Medline: 24994699]
151. Lehmann T, Heß M, Wanner G, Melzer RR. Dissecting a neuron network: FIB-SEM-based 3D-reconstruction of the visual neuropils in the sea spider Achelia langi (Dohrn, 1881) (Pycnogonida). BMC Biol 2014 Aug 13;12:59 [FREE Full text] [doi: 10.1186/s12915-014-0059-3] [Medline: 25285383]
152. Kucharzewski C, Raselimanana AP, Wang C, Glaw F. A taxonomic mystery for more than 150 years: Identity, systematic position and Malagasy origin of the snake Elapotinus picteti Jan, 1862, and synonymy of Exallodontophis Cadle, 1999 (Serpentes: Lamprophiidae). Zootaxa 2014 Aug 14;3852(2):179-202. [Medline: 25284393]
153. Brenzinger B, Wilson NG, Schrödl M. Microanatomy of shelled Koloonella cf. minutissima (Laseron, 1951) (Gastropoda: 'lower' Heterobranchia: Murchisonellidae) does not contradict a sister-group relationship with enigmatic Rhodopemorpha slugs. J Mollus Stud 2014 Jun 27;80(5):518-540. [doi: 10.1093/mollus/eyu036]
154. Scherz MD, Ruthensteiner B, Vieites DR, Vences M, Glaw F. Two new microhylid frogs of the genus Rhombophryne with superciliary spines from the Tsaratanana Massif in Northern Madagascar. Herpetologica 2015 Dec;71(4):310-321. [doi: 10.1655/HERPETOLOGICA-D-14-00048]
155. Schwaha TF, Handschuh S, Redl E, Wanninger A. Insights into the organization of plumatellid larvae (lophotrochozoa, Bryozoa) by means of 3D-imaging and confocal microscopy. J Morphol 2015 Jan;276(1):109-120. [doi: 10.1002/jmor.20326] [Medline: 25278218]
156. Grabe V, Strutz A, Baschwitz A, Hansson BS, Sachse S. Digital in vivo 3D atlas of the antennal lobe of Drosophila melanogaster. J Comp Neurol 2015 Feb 15;523(3):530-544. [doi: 10.1002/cne.23697] [Medline: 25327641]
157. Sharp AC, Trusler PW. Morphology of the jaw-closing musculature in the common wombat (Vombatus ursinus) using digital dissection and magnetic resonance imaging. PLoS One 2015;10(2):e0117730 [FREE Full text] [doi: 10.1371/journal.pone.0117730] [Medline: 25707001]
158. Christensen CB, Lauridsen H, Christensen-Dalsgaard J, Pedersen M, Madsen PT. Better than fish on land? Hearing across metamorphosis in salamanders. Proc Biol Sci 2015 Mar 07;282(1802) [FREE Full text] [doi: 10.1098/rspb.2014.1943] [Medline: 25652830]
159. Wild E, Wollesen T, Haszprunar G, Heß M. Comparative 3D microanatomy and histology of the eyes and central nervous systems in coleoid cephalopod hatchlings. Org Divers Evol 2014 Dec 12;15(1):37-64. [doi: 10.1007/s13127-014-0184-4]

XSL•FO
RenderX

160. Chen C, Copley JT, Linse K, Rogers AD, Sigwart JD. The heart of a dragon: 3D anatomical reconstruction of the 'scaly-foot gastropod' (Mollusca: Gastropoda: Neomphalina) reveals its extraordinary circulatory system. Front Zool 2015;12:13 [FREE Full text] [doi: 10.1186/s12983-015-0105-1] [Medline: 26085836]
161. Kelder TP, Vicente-Steijn R, Harryvan TJ, Kosmidis G, Gittenberger-de Groot AC, Poelmann RE, et al. The sinus venosus myocardium contributes to the atrioventricular canal: potential role during atrioventricular node development? J Cell Mol Med 2015 Jun;19(6):1375-1389 [FREE Full text] [doi: 10.1111/jcmm.12525] [Medline: 25752780]
162. Soffers JH, Hikspoors JP, Mekonen HK, Koehler SE, Lamers WH. The growth pattern of the human intestine and its mesentery. BMC Dev Biol 2015 Aug 22;15:31 [FREE Full text] [doi: 10.1186/s12861-015-0081-x] [Medline: 26297675]
163. Vicente-Steijn R, Scherptong RW, Kruithof BP, Duim SN, Goumans MJ, Wisse LJ, et al. Regional differences in WT-1 and Tcf21 expression during ventricular development: implications for myocardial compaction. PLoS One 2015;10(9):e0136025 [FREE Full text] [doi: 10.1371/journal.pone.0136025] [Medline: 26390289]
164. Bonsmann A, Stoffel MH, Burkhart M, Hatt JM. Anatomical atlas of the quail's ear (Coturnix coturnix). Anat Histol Embryol 2016 Oct;45(5):399-404. [doi: 10.1111/ahe.12198] [Medline: 26396065]
165. Razafimahatratra B, Wang C, Mori A, Glaw F. Potential envenomation by the aglyphous pseudoxyrhophiine snake Leioheterodon madagascariensis and description of its dentition. J Venom Anim Toxins Incl Trop Dis 2015;21:47 [FREE Full text] [doi: 10.1186/s40409-015-0047-2] [Medline: 26594226]
166. van de Kamp T, Cecilia A, dos Santos Rolo T, Vagovič P, Baumbach T, Riedel A. Comparative thorax morphology of death-feigning flightless cryptorhynchine weevils (Coleoptera: Curculionidae) based on 3D reconstructions. Arthropod Struct Dev 2015 Nov;44(6):509-523. [doi: 10.1016/j.asd.2015.07.004] [Medline: 26259678]
167. Sumner-Rooney LH, Schrödl M, Lodde-Bensch E, Lindberg DR, Heß M, Brennan GP, et al. A neurophylogenetic approach provides new insight to the evolution of Scaphopoda. Evol Dev 2015;17(6):337-346. [doi: 10.1111/ede.12164] [Medline: 26487042]
168. D'Amato G, Luxán G, del Monte-Nieto G, Martínez-Poveda B, Torroja C, Walter W, et al. Sequential Notch activation regulates ventricular chamber development. Nat Cell Biol 2016 Jan;18(1):7-20 [FREE Full text] [doi: 10.1038/ncb3280] [Medline: 26641715]
169. Loll B, Fabian H, Huser H, Hee CS, Ziegler A, Uchanska-Ziegler B, et al. Increased conformational flexibility of HLA-B*27 subtypes associated with ankylosing spondylitis. Arthritis Rheumatol 2016 Dec;68(5):1172-1182 [FREE Full text] [doi: 10.1002/art.39567] [Medline: 26748477]
170. Felix PM, Gonçalves A, Vicente JR, Fonseca PJ, Amorim MC, Costa JL, et al. Optical micro-tomography “OPenT” allows the study of large toadfish Halobatrachus didactylus embryos and larvae. Mech Dev 2016 Dec;140:19-24 [FREE Full text] [doi: 10.1016/j.mod.2016.03.001] [Medline: 27000637]
171. Rybak J, Talarico G, Ruiz S, Arnold C, Cantera R, Hansson BS. Synaptic circuitry of identified neurons in the antennal lobe of Drosophila melanogaster. J Comp Neurol 2016 Jun 15;524(9):1920-1956. [doi: 10.1002/cne.23966] [Medline: 26780543]
172. Fischer G, Sarnat EM, Economo EP. Revision and microtomography of the Pheidole knowlesi group, an endemic ant radiation in Fiji (Hymenoptera, Formicidae, Myrmicinae)Myrmicinae). PLoS One 2016;11(7):e0158544 [FREE Full text] [doi: 10.1371/journal.pone.0158544] [Medline: 27462877]
173. Haszprunar G, Kunze T, Brückner M, Heß M. Towards a sound definition of Skeneidae (Mollusca, Vetigastropoda): 3D interactive anatomy of the type species, Skenea serpuloides (Montagu, 1808) and comments on related taxa. Org Divers Evol 2016 Jan 14;16(3):577-595. [doi: 10.1007/s13127-015-0260-4]
174. Neusser TP, Jörger KM, Lodde-Bensch E, Strong EE, Schrödl M. The unique deep sea-land connection: interactive 3D visualization and molecular phylogeny ofn. sp. (Bathyhedylidae n. fam.)-the first panpulmonate slug from bathyal zones. PeerJ 2016;4:e2738 [FREE Full text] [doi: 10.7717/peerj.2738] [Medline: 27957391]
175. Scherz MD, Hawlitschek O, Andreone F, Rakotoarison A, Vences M, Glaw F. A review of the taxonomy and osteology of the Rhombophryne serratopalpebrosa species group (Anura: Microhylidae) from Madagascar, with comments on the value of volume rendering of micro-CT data to taxonomists. Zootaxa 2017 Jun 06;4273(3):301-340. [doi: 10.11646/zootaxa.4273.3.1]
176. Wu Y, Dabhoiwala NF, Hagoort J, Tan LW, Zhang SX, Lamers WH. Architectural differences in the anterior and middle compartments of the pelvic floor of young-adult and postmenopausal females. J Anat 2017 May;230(5):651-663. [doi: 10.1111/joa.12598] [Medline: 28299781]
177. Lambert SM, Hutter CR, Scherz MD. Diamond in the rough: a new species of fossorial diamond frog (Rhombophryne) from Ranomafana National Park, southeastern Madagascar. ZSE 2017 Feb 24;93(1):143-155. [doi: 10.3897/zse.93.10188]
178. Chen C, Uematsu K, Linse K, Sigwart JD. By more ways than one: Rapid convergence at hydrothermal vents shown by 3D anatomical reconstruction of Gigantopelta (Mollusca: Neomphalina). BMC Evol Biol 2017 Dec 01;17(1):62 [FREE Full text] [doi: 10.1186/s12862-017-0917-z] [Medline: 28249568]
179. Klinkhamer AJ, Wilhite DR, White MA, Wroe S. Digital dissection and three-dimensional interactive models of limb musculature in the Australian estuarine crocodile (Crocodylus porosus). PLoS One 2017;12(4):e0175079 [FREE Full text] [doi: 10.1371/journal.pone.0175079] [Medline: 28384201]

XSL•FO
RenderX

180. Herzog H, Klein B, Ziegler A. Form and function of the teleost lateral line revealed using three-dimensional imaging and computational fluid dynamics. J R Soc Interface 2017 May;14(130). [doi: 10.1098/rsif.2016.0898] [Medline: 28468922]
181. Smith TD, McMahon MJ, Millen ME, Llera C, Engel SM, Li L, et al. Growth and development at the sphenoethmoidal junction in perinatal primates. Anat Rec (Hoboken) 2017 Dec;300(12):2115-2137. [doi: 10.1002/ar.23630] [Medline: 28667704]
182. Dreyer N, Høeg JT, Heß M, Sørensen S, Spremberg U, Yusa Y. When dwarf males and hermaphrodites copulate: first record of mating behaviour in a dwarf male using the androdioecious barnacle Scalpellum scalpellum (Crustacea: Cirripedia: Thoracica). Org Divers Evol 2018 Mar;18(1):115-123. [doi: 10.1007/s13127-017-0349-z]
183. Fredericksen MA, Zhang Y, Hazen ML, Loreto RG, Mangold CA, Chen DZ, et al. Three-dimensional visualization and a deep-learning model reveal complex fungal parasite networks in behaviorally manipulated ants. Proc Natl Acad Sci U S A 2017 Nov 21;114(47):12590-12595 [FREE Full text] [doi: 10.1073/pnas.1711673114] [Medline: 29114054]
184. de Bakker BS, de Bakker HM, Soerdjbalie-Maikoe V, Dikkers FG. The development of the human hyoid-larynx complex revisited. Laryngoscope 2017 Dec 08. [doi: 10.1002/lary.26987] [Medline: 29219191]
185. Betts MW, Maschner HD, Schou CD, Schlader R, Holmes J, Clement N, et al. Virtual zooarchaeology: building a web-based reference collection of northern vertebrates for archaeofaunal research and education. Journal of Archaeological Science 2011 Apr;38(4):755.e1-755.e9. [doi: 10.1016/j.jas.2010.06.021]
186. Pingault JB, Côté SM, Lacourse E, Galéra C, Vitaro F, Tremblay RE. Childhood hyperactivity, physical aggression and criminality: a 19-year prospective population-based study. PLoS One 2013;8(5):e62594 [FREE Full text] [doi: 10.1371/journal.pone.0062594] [Medline: 23658752]
187. Poelmann RE, Gittenberger-de Groot AC, Vicente-Steijn R, Wisse LJ, Bartelings MM, Everts S, et al. Evolution and development of ventricular septation in the amniote heart. PLoS One 2014;9(9):e106569 [FREE Full text] [doi: 10.1371/journal.pone.0106569] [Medline: 25192012]
188. Li Z, Clarke JA. New insight into the anatomy of the hyolingual apparatus of Alligator mississippiensis and implications for reconstructing feeding in extinct archosaurs. J Anat 2015 Jul;227(1):45-61 [FREE Full text] [doi: 10.1111/joa.12320] [Medline: 26018316]
189. Charles JP, Cappellari O, Spence AJ, Hutchinson JR, Wells DJ. Musculoskeletal geometry, muscle architecture and functional specialisations of the mouse hindlimb. PLoS One 2016;11(4):e0147669 [FREE Full text] [doi: 10.1371/journal.pone.0147669] [Medline: 27115354]
190. Porter WR, Sedlmayr JC, Witmer LM. Vascular patterns in the heads of crocodilians: blood vessels and sites of thermal exchange. J Anat 2016 Dec;229(6):800-824. [doi: 10.1111/joa.12539] [Medline: 27677246]
191. Shin DS, Shim YJ, Kim BC. Sectioned images and surface models of a cadaver head with reference to botulinum neurotoxin injection. Folia Morphol (Warsz) 2018 Jan 18 [FREE Full text] [doi: 10.5603/FM.a2018.0005] [Medline: 29345721]
192. Husch A, Petersen MV, Gemmar P, Goncalves J, Sunde N, Hertel F. Post-operative deep brain stimulation assessment: Automatic data integration and report generation. Brain Stimul 2018 Feb 01 [FREE Full text] [doi: 10.1016/j.brs.2018.01.031] [Medline: 29429953]
193. Shin DS, Shim YJ, Kim BC. Sectioned images and 3D models of a cadaver head with reference to dermal filler injection. Ann Anat 2018 May;217:34-39. [doi: 10.1016/j.aanat.2018.02.001] [Medline: 29481857]
194. Bicknell RD, Klinkhamer AJ, Flavel RJ, Wroe S, Paterson JR. A 3D anatomical atlas of appendage musculature in the chelicerate arthropod Limulus polyphemus. PLoS One 2018;13(2):e0191400 [FREE Full text] [doi: 10.1371/journal.pone.0191400] [Medline: 29444161]
195. van Wijk B, van den Berg G, Abu-Issa R, Barnett P, van der Velden S, Schmidt M, et al. Epicardium and myocardium separate from a common precursor pool by crosstalk between bone morphogenetic protein- and fibroblast growth factor-signaling pathways. Circ Res 2009 Aug 28;105(5):431-441 [FREE Full text] [doi: 10.1161/CIRCRESAHA.109.203083] [Medline: 19628790]
196. Shin DS, Jang HG, Park JS, Park HS, Lee S, Chung MS. Accessible and informative sectioned images and surface models of a cadaver head. J Craniofac Surg 2012 Jul;23(4):1176-1180. [doi: 10.1097/SCS.0b013e31825657d8] [Medline: 22801119]
197. Prats-Galino A, Reina MA, Mavar-Haramija M, Puigdellivol-Sánchez A, Juanes Mendez JA, de Andres JA. 3D interactive model of lumbar spinal structures of anesthetic interest. Clin Anat 2015 Mar;28(2):205-212. [doi: 10.1002/ca.22479] [Medline: 25352014]
198. Shin DS, Jang HG, Hwang SB, Har DH, Moon YL, Chung MS. Two-dimensional sectioned images and three-dimensional surface models for learning the anatomy of the female pelvis. Anat Sci Educ 2013;6(5):316-323. [doi: 10.1002/ase.1342] [Medline: 23463707]
199. Chung BS, Ahn YH, Park JS. Ten triangles around cavernous sinus for surgical approach, described by schematic diagram and three dimensional models with the sectioned images. J Korean Med Sci 2016 Sep;31(9):1455-1463 [FREE Full text] [doi: 10.3346/jkms.2016.31.9.1455] [Medline: 27510391]
200. de Bakker BS, de Jong KH, Hagoort J, de Bree K, Besselink CT, de Kanter FE, et al. An interactive three-dimensional digital atlas and quantitative database of human development. Science 2016 Dec 25;354(6315):aag0053. [doi: 10.1126/science.aag0053] [Medline: 27884980]

XSL•FO
RenderX

201. Park HS, Chung MS, Shin DS, Jung YW, Park JS. Accessible and informative sectioned images, color-coded images, and surface models of the ear. Anat Rec (Hoboken) 2013 Aug;296(8):1180-1186 [FREE Full text] [doi: 10.1002/ar.22719] [Medline: 23713007]
202. Park HS, Shin DS, Cho DH, Jung YW, Park JS. Improved sectioned images and surface models of the whole dog body. Ann Anat 2014 Sep;196(5):352-359. [doi: 10.1016/j.aanat.2014.05.036] [Medline: 24986152]
203. Kim BC, Chung MS, Park HS, Shin DS, Park JS. Accessible and informative sectioned images and surface models of the maxillofacial area for orthognathic surgery. Folia Morphol (Warsz) 2015;74(3):346-351. [doi: 10.5603/FM.2015.0052] [Medline: 26339816]
204. Shin DS, Lee S, Park HS, Lee SB, Chung MS. Segmentation and surface reconstruction of a cadaver heart on Mimics software. Folia Morphol (Warsz) 2015;74(3):372-377. [doi: 10.5603/FM.2015.0056] [Medline: 26339820]
205. Shin DS, Kim HJ, Kim BC. Sectioned images and surface models of a cadaver for understanding the dorsalis pedis flap. J Craniofac Surg 2015 Jul;26(5):1656-1659. [doi: 10.1097/SCS.0000000000001618] [Medline: 26079120]
206. Park JS, Jung YW. Software for browsing sectioned images of a dog body and generating a 3D model. Anat Rec (Hoboken) 2016 Jan;299(1):81-87 [FREE Full text] [doi: 10.1002/ar.23200] [Medline: 26219434]
207. Reina MA, Lirk P, Puigdellívol-Sánchez A, Mavar M, Prats-Galino A. Human lumbar ligamentum flavum anatomy for epidural anesthesia: reviewing a 3D MR-based interactive model and postmortem samples. Anesth Analg 2016 Mar;122(3):903-907. [doi: 10.1213/ANE.0000000000001109] [Medline: 26891398]
208. Shin DS, Kim HJ, Kim BC. Sectioned images and surface models of a cadaver for understanding the free vascularised anterior rib flap. Folia Morphol (Warsz) 2017;76(1):117-122. [doi: 10.5603/FM.a2016.0035] [Medline: 27830889]
209. Jang HG, Chung MS, Shin DS. Portable Document Format file containing the surface models to learn the stereoscopic shape of foot muscles. Int J Morphol 2015 Dec;33(4):1287-1292. [doi: 10.4067/S0717-95022015000400016]
210. Shin DS, Chung MS. Virtual movement of the ankle and subtalar joints using cadaver surface models. Int J Morphol 2015 Sep;33(3):888-894. [doi: 10.4067/S0717-95022015000300014]
211. Chung BS, Shin DS, Brown P, Choi J, Chung MS. Virtual dissection table including the visible Korean images, complemented by free software of the same data. Int J Morphol 2015 Jun;33(2):440-445. [doi: 10.4067/S0717-95022015000200006]
212. Park HS, Chung MS, Shin DS, Jung YW, Park JS. Whole courses of the oculomotor, trochlear, and abducens nerves, identified in sectioned images and surface models. Anat Rec (Hoboken) 2015 Feb;298(2):436-443 [FREE Full text] [doi: 10.1002/ar.23048] [Medline: 25212480]
213. Shin DS, Kwon K, Shin B, Park HS, Lee S, Lee S, et al. Surface models and gradually stripped volume model to explore the foot muscles. Anatomy 2015 Apr 30;9(1):19-25. [doi: 10.2399/ana.14.052]
214. Chung BS, Chung MS, Park JS. Six walls of the cavernous sinus identified by sectioned images and three-dimensional models: anatomic report. World Neurosurg 2015 Aug;84(2):337-344. [doi: 10.1016/j.wneu.2015.03.049] [Medline: 25839400]
215. Chung BS, Park JS, Jang HG, Chung MS. Software to browse the pictures of two knees in diverse states of dissection, flexion and rotation. Int J Morphol 2015 Sep;33(3):1009-1015. [doi: 10.4067/S0717-95022015000300032]
216. Chung BS, Chung MS, Park HS, Shin B, Kwon K. Colonoscopy tutorial software made with a cadaver's sectioned images. Ann Anat 2016 Nov;208:19-23. [doi: 10.1016/j.aanat.2016.06.010] [Medline: 27475426]
217. Chung BS, Kwon K, Shin BS, Chung MS. Surface models and gradually peeled volume model to explore hand structures. Ann Anat 2017 May;211:202-206. [doi: 10.1016/j.aanat.2017.02.002] [Medline: 28274804]
218. Park HS, Choi DH, Park JS. Improved sectioned images and surface models of the whole female body. Int J Morphol 2015 Dec;33(4):1323-1332. [doi: 10.4067/S0717-95022015000400022]
219. Chung BS, Chung MS, Shin BS, Kwon K. Three software tools for viewing sectional planes, volume models, and surface models of a cadaver hand. J Korean Med Sci 2018 Feb 19;33(8):e64 [FREE Full text] [doi: 10.3346/jkms.2018.33.e64] [Medline: 29441756]
220. Valera-Melé M, Puigdellívol-Sánchez A, Mavar-Haramija M, Juanes-Méndez JA, San-Román L, de Notaris M, et al. A novel and freely available interactive 3D model of the internal carotid artery. J Med Syst 2018 Mar 05;42(4):72. [doi: 10.1007/s10916-018-0919-4] [Medline: 29508089]
221. Warmann SW, Schenk A, Schaefer JF, Ebinger M, Blumenstock G, Tsiflikas I, et al. Computer-assisted surgery planning in children with complex liver tumors identifies variability of the classical Couinaud classification. J Pediatr Surg 2016 Nov;51(11):1801-1806. [doi: 10.1016/j.jpedsurg.2016.05.018] [Medline: 27289416]
222. Barnes DG, Fluke CJ, Bourke PD, Parry OT. An advanced, three-dimensional plotting library for astronomy. Publ Astron Soc Aust 2013 Mar 5;23(02):82-93. [doi: 10.1071/AS06009]
223. Swinburne University of Technology. S2PLOT: A three-dimensional plotting library URL: http://astronomy.swin.edu.au/s2plot/index.php?title=S2PLOT [accessed 2018-03-03] [WebCite Cache ID 6xdskoYtn]
224. The LaTeX Project. LaTeX - A document preparation system URL: https://www.latex-project.org [accessed 2018-03-03] [WebCite Cache ID 6xdseFf7s]
225. Grahn A. CTAN Comprehensive Tex Archive Network. movie15 – Multimedia inclusion package URL: https://ctan.org/pkg/movie15 [accessed 2018-03-03] [WebCite Cache ID 6xdsVN39p]
226. Asymptote. Asymptote - SourceForge URL: http://asymptote.sourceforge.net [accessed 2018-03-03] [WebCite Cache ID 6xdstUnRp]

XSL•FO
RenderX

227. Haru free PDF library. URL: http://libharu.org/ [accessed 2018-03-03] [WebCite Cache ID 6xdt1s7rK]
228. Ritter F, Boskamp T, Homeyer A, Laue H, Schwier M, Link F, et al. Medical image analysis. IEEE Pulse 2011 Nov;2(6):60-70. [doi: 10.1109/MPUL.2011.942929] [Medline: 22147070]
229. MeVis Medical Solutions AG and Fraunhofer MEVIS. MeVisLab. URL: https://www.mevislab.de/ [accessed 2018-03-03] [WebCite Cache ID 6xduIVHna]
230. de Bakker BS. 3D Atlas of Human Embryology. URL: https://www.3dembryoatlas.com/ [accessed 2018-03-03] [WebCite Cache ID 6xdtZCf9G]
231. KISTI. Visible Korean. Overview of the Visible Korean Project URL: http://vkh3.kisti.re.kr/?q=node/24 [accessed 2018-03-03] [WebCite Cache ID 6xdtfOivE]
232. Springob CM, Magoulas C, Colless M, Mould J, Erdogdu P, Jones DH, et al. The 6dF Galaxy Survey: peculiar velocity field and cosmography. Mon Not R Astron Soc 2014;445(3):2677-2697. [doi: 10.1093/mnras/stu1743]
233. Lautenschlager S, Butler RJ. Neural and endocranial anatomy of Triassic phytosaurian reptiles and convergence with fossil and modern crocodylians. PeerJ 2016;4:e2251 [FREE Full text] [doi: 10.7717/peerj.2251] [Medline: 27547557]
234. Zanno LE, Drymala S, Nesbitt SJ, Schneider VP. Early crocodylomorph increases top tier predator diversity during rise of dinosaurs. Sci Rep 2015 Mar 19;5:9276 [FREE Full text] [doi: 10.1038/srep09276] [Medline: 25787306]
235. Vasilyev V. Towards interactive 3D graphics in chemistry publications. Theor Chem Acc 2010;125(3-6):173-176. [doi: 10.1007/s00214-009-0636-7.x]

Abbreviations

2D: two-dimensional
3D: three-dimensional
ECMA: European Computer Manufacturers Association
ISO: International Organization for Standardization
MiB: mebibyte, (binary) megabytes
PDF: Portable Document Format
PMI: product manufacturing information
PRC: Product Representation Compact
U3D: Universal 3D

Edited by G Eysenbach; submitted 03.03.18; peer-reviewed by M Puttaroo, J Veenland; comments to author 26.04.18; revised version received 07.05.18; accepted 24.05.18; published 07.08.18

Please cite as:
Newe A, Becker L
Three-Dimensional Portable Document Format (3D PDF) in Clinical Communication and Biomedical Sciences: Systematic Review of Applications, Tools, and Protocols
JMIR Med Inform 2018;6(3):e10295
URL: http://medinform.jmir.org/2018/3/e10295/
doi:10.2196/10295
PMID:

©Axel Newe, Linda Becker. Originally published in JMIR Medical Informatics (http://medinform.jmir.org), 07.08.2018. This is an open-access article distributed under the terms of the Creative Commons Attribution License (https://creativecommons.org/licenses/by/4.0/), which permits unrestricted use, distribution, and reproduction in any medium, provided the original work, first published in JMIR Medical Informatics, is properly cited. The complete bibliographic information, a link to the original publication on http://medinform.jmir.org/, as well as this copyright and license information must be included.

XSL•FO
RenderX
